# Spatio-Temporal Patterns of Demyelination and Remyelination in the Cuprizone Mouse Model

**DOI:** 10.1371/journal.pone.0152480

**Published:** 2016-04-07

**Authors:** Ian Tagge, Audrey O’Connor, Priya Chaudhary, Jim Pollaro, Yosef Berlow, Megan Chalupsky, Dennis Bourdette, Randy Woltjer, Mac Johnson, William Rooney

**Affiliations:** 1 Advanced Imaging Research Center, Oregon Health & Science University, 3181 SW Sam Jackson Park Rd, Portland, OR 97239, United States of America; 2 Biomedical Engineering, Oregon Health & Science University, 3181 SW Sam Jackson Park Rd, Portland, OR 97239, United States of America; 3 Neurology, Oregon Health & Science University, 3181 SW Sam Jackson Park Rd, Portland, OR 97239, United States of America; 4 Pathology, Oregon Health & Science University, 3181 SW Sam Jackson Park Rd, Portland, OR 97239, United States of America; 5 Portland VA Medical Center, 3710 SW US Veterans Hospital Rd, Portland, OR 97239, United States of America; 6 Vertex Pharmaceuticals Incorporated, 50 Northern Ave, Boston, MA 02210, United States of America; Hannover Medical School, GERMANY

## Abstract

Cuprizone administration in mice provides a reproducible model of demyelination and spontaneous remyelination, and has been useful in understanding important aspects of human disease, including multiple sclerosis. In this study, we apply high spatial resolution quantitative MRI techniques to establish the spatio-temporal patterns of acute demyelination in C57BL/6 mice after 6 weeks of cuprizone administration, and subsequent remyelination after 6 weeks of post-cuprizone recovery. MRI measurements were complemented with Black Gold II stain for myelin and immunohistochemical stains for associated tissue changes. Gene expression was evaluated using the Allen Gene Expression Atlas. Twenty-five C57BL/6 male mice were split into control and cuprizone groups; MRI data were obtained at baseline, after 6 weeks of cuprizone, and 6 weeks post-cuprizone. High-resolution (100μm isotropic) whole-brain coverage magnetization transfer ratio (MTR) parametric maps demonstrated concurrent caudal-to-rostral and medial-to-lateral gradients of MTR decrease within corpus callosum (CC) that correlated well with demyelination assessed histologically. Our results show that demyelination was not limited to the midsagittal line of the corpus callosum, and also that opposing gradients of demyelination occur in the lateral and medial CC. T_2_-weighted MRI gray/white matter contrast was strong at baseline, weak after 6 weeks of cuprizone treatment, and returned to a limited extent after recovery. MTR decreases during demyelination were observed throughout the brain, most clearly in callosal white matter. Myelin damage and repair appear to be influenced by proximity to oligodendrocyte progenitor cell populations and exhibit an inverse correlation with myelin basic protein gene expression. These findings suggest that susceptibility to injury and ability to repair vary across the brain, and whole-brain analysis is necessary to accurately characterize this model. Whole-brain parametric mapping across time is essential for gaining a real understanding of disease processes in-vivo. MTR increases in healthy mice throughout adolescence and adulthood were observed, illustrating the need for appropriate age-matched controls. Elucidating the unique and site-specific demyelination in the cuprizone model may offer new insights into in mechanisms of both damage and repair in human demyelinating diseases.

## Introduction

Cuprizone [bis-cyclohexanone-oxaldihydrazone] is a low molecular weight copper chelator that induces reversible demyelination in both gray and white matter in the murine brain when added to chow in low concentrations for short periods. First described as a neurotoxin in rodents in the 1960’s, cuprizone reliably produces toxic effects including demyelination, hydrocephalus, and astrogliosis.[1,2] The cuprizone mouse captures some aspects of multiple sclerosis (MS), providing a model of demyelination and spontaneous remyelination. Non-focal demyelinating lesions in this model occur in the presence of microglial activation and oligodendrocyte apoptosis without lymphocytic infiltration, which can occur in some MS lesions.[3,4]

While cuprizone administration in the mouse has become a common approach used to study demyelination and remyelination processes relevant to human disease, the mechanism of cuprizone action and subsequent oligodendrocyte death is not well understood. Recent reports suggest cuprizone does not accumulate in the brain;[5] rather, cuprizone toxicity extensively modifies copper and zinc distribution in the brain, resulting in mitochondrial dysfunction that leads to demyelination.[6–9] Spatial heterogeneity in brain pathology in the cuprizone model has been demonstrated,[10–15] and the mechanism of demyelination may vary across structures. Because histological analyses are invasive and time-intensive, non-invasive imaging techniques are well suited to complement histology and provide a more comprehensive perspective of pathophysiology, particularly with respect to longitudinal studies. Careful histological analyses are important to validate emerging quantitative and semi-quantitative in-vivo imaging techniques.

Several magnetic resonance imaging (MRI) based methods of non-invasively quantifying demyelination in-vivo in the cuprizone mouse model have been explored.[16–21] Magnetization Transfer (MT) has been widely used as a fast and precise measurement capable of semi-quantitative estimation of macromolecular content by calculating the MT ratio (MTR). Myelin content correlates with MTR, but, axonal density and other tissue components can also influence MTR values.[22]

Due to signal-to-noise (SNR) limitations, particularly when imaging small rodents, in-vivo MRI experiments tend to utilize single- or multi-slice acquisitions with thick slices (0.5–1.0mm) and limited coverage.[13,18,20,23] Mouse brains are roughly 10mm across compared to 120mm in humans. A voxel size of 100μm^3^ or less is thus required to achieve resolution comparable to the 1mm^3^ voxel size in human neuroimaging. Some recent work has obtained 3D whole-brain MT images with good resolution (200x200x230μm^3^[12] or 117μm isotropic[19,24]), although results presented included only either single-slice or region-of-interest (ROI) analysis. While ROI analysis is useful for boosting SNR and performing coarse regional evaluations, it necessarily introduces exaggerated partial-volume dilution and obscures fine regional and structural variations. This latter point is of particular interest because pathology and morphology are known to be highly heterogeneous both regionally and across animals in the cuprizone model.[10–13,15,25–27]

In this study we investigated non-invasive methods of characterizing demyelination and remyelination in-vivo. We employed T_2_-weighted and magnetization transfer imaging sequences, established semi-quantitative MRI techniques designed to achieve whole-brain coverage with exceptional spatial resolution (100μm isotropic), to elucidate the spatial distribution of acute cuprizone-induced demyelination, and subsequent remyelination, in adult C57BL/6 male mice. Gold-standard histological analyses were used to evaluate the extent to which MTR was a specific measure of myelin content in-vivo. We thus confirm and expand upon earlier work[11,12] as we present the first comprehensive overview of spatially varying cuprizone-induced demyelination in the mouse corpus callosum (CC) and external capsule (EC).

## Materials and Methods

This study was specifically reviewed and approved by the Oregon Health & Science University (OHSU) Institutional Animal Care and Use Committee (IACUC), as protocol IS00001282. All animal handling, care and treatment was carried out in strict accordance with the OHSU IACUC regulations. C57BL/6 mice were obtained from Charles River. Mice (n = 11 controls, 14 cuprizone) were studied longitudinally for up to twelve weeks. Mice were monitored daily for signs of distress including lethargy, hunching, and self-mutilation. Each mouse was weighed weekly and values recorded. Sustained weight loss, failure to recover from anesthesia, and general poor health were defined as humane end points. A malocclusion led to malnourishment and eventual humane euthanasia in one mouse during the acclimation period. One cuprizone-treated mouse died of unknown causes the day after successfully recovering from Week 6 MRI. No other adverse events occurred in this study.

Twenty-six male C57BL/6 mice were received at 4 weeks of age and singly housed on-site under normal light/dark cycle conditions. After a 3-week acclimation period, 14 mice began a 0.2% (wt/wt) cuprizone diet (Sigma-Aldrich, St Louis, MO) with free access to food. All animals were given pelletized chow for the duration of the study (Purina Mills, LLC, TestDiet division). MRI examinations were performed longitudinally at three time points: (1) immediately prior to initiation of cuprizone diet (age 7 weeks; Baseline), (2) at the end of treatment (age 13 weeks; Week 6) and (3) 6 weeks later (age 19 weeks; Week 12 for controls, Week 6+6 for cuprizone animals: i.e., 6 weeks on cuprizone and 6 weeks normal chow post-cuprizone administration). After 6 weeks of treatment a subgroup (n = 9 treatment and 6 age-matched controls) were sacrificed immediately following MRI for histological analyses. The remaining mice were returned to a normal diet for 6 weeks of recovery at which time a final MRI was obtained and all remaining subjects were sacrificed for histology. Euthanasia was accomplished by isoflurane overdose followed by cervical dislocation.

### MRI

MRI data were collected using an 11.75 Tesla (T) instrument (Bruker Biospin, Billerica MA) equipped with a high-performance gradient coil (9 cm inner diameter), and radiofrequency (RF) volume coil transmitter and surface receiver. The RF coils are sequentially detuned to reduce interactions between the transmitter and receiver RF coils. The surface coil was custom-built and had an oval geometry with 1.2cm long axis and 0.8cm short axis. Prior to image acquisition, a gradient-based magnetic field optimization routine was performed to adjust electrical currents in first, second, and third-order room-temperature shims. Mice were initially sedated via an intraperitoneal injection of a xylazine/ketamine cocktail, and then maintained with 1–2% isofluorane, adjusted to maintain respiration (approx. 90±10 breaths/min), in 100% oxygen for the duration of the MRI (approx. 2.5hrs). Rectal temperature and respiration rate were monitored throughout the study using a small animal physiological monitor (SA Instruments, Inc., Stony Brook, NY). The magnet bore was heated with forced warm air using the rectal thermometer as controller. Core body temperature was maintained at 37° ± 1°C. Custom-built head-holders restrained the sedated animal to minimize respiration-induced motion artifacts.

A two-dimensional multislice T_2_-weighted RARE sequence was acquired (refocus flip angle (FA) 180°, field-of-view (FOV) 1.3cmx1.6cm (132x160 matrix, 100x100μm^2^ in-plane resolution), 25 0.5mm thick coronal slices). Magnetization transfer (MT) MRI data were acquired using whole-brain 3D gradient-recalled echo (GRE) sequences with 2.5ms echo time (TE), 30ms recycle time (TR), 10° FA, and a pulsed MT saturation pulse of Gauss shape, 20ms duration, 0.01ms interpulse delay, 137 Hz bandwidth. In an effort to remain reasonably consistent with other work in humans[28–30] and mice[12,13,19,24,31,32] we collected MT images with B_1_ field strength: 7.8μT (corresponding to 1000° effective FA), and offset frequency Δ: +4kHz. All 3D GRE MT sequences were acquired at 100μm isotropic resolution and full brain coverage (field-of-view 1.92cm x 1.44cm x 0.96cm). An identical reference magnitude image, M_0_, was collected with no MT saturation pulse.

### Tissue processing

Brains were extracted whole within 10 minutes of sacrifice, immediately immersed in 4% paraformaldehyde and microwaved for 75 min using the PELCO BioWave^®^ Pro (Ted Pella, Inc., Redding, CA). The entire brain was sectioned into 30μm slices using a Leica VT1000s Vibratome. About 20 representative sections between bregma -2mm and bregma +1.5mm were stained with Black Gold II and imaged using a Zeiss AxioImager M2 bright field microscope.

#### Myelin staining and analysis

Brain sections (30μm) were stained with Black Gold II (AG105, Millipore Corp, Billerica, MA) according to manufacturer’s instructions. Briefly, the sections were dehydrated for 60–90 minutes on a slide warmer and then rehydrated with purified water (“Milli-Q” water purified using a Millipore system). Pre-warmed Black Gold II solution was added onto sections and incubated at 60°C. The average incubation time was 15 min. The slides were rinsed with Milli-Q water twice. Pre-warmed 1% sodium thiosulfate was added to the slides and incubated for 3 min. The slides were rinsed thrice with Milli-Q water before 3-minute incubation with cresyl violet stain. Sections were rinsed again and dehydrated using a series of gradated alcohols and finally in a xylene substitute for 2 min and coverslipped with mounting media. The area of demyelination in the corpus callosum was manually measured using MetaMorph (ver 7.7.5, Molecular Devices, CA) and represented as percent demyelination. Analysis of demyelinated area was restricted to the MRI-visible extents of the corpus callosum.

#### Immunohistochemical studies

Fixed brain tissue was used to prepare 6-micron paraffin-embedded sections. After deparaffinization and antigen retrieval (5 min treatment at room temperature with 95% formic acid, followed by 30 min incubation in citrate buffer, pH 6.0, at 90 degrees C. Tissue sections were blocked with 5% nonfat dry milk in phosphate-buffered saline and labeled with antibodies to GFAP (G9269 from Sigma, St. Louis, MO), microglia (Iba1 from Wako USA, Richmonda, VA), and PDGFR-alpha (R&D systems, Minneapolis, MN). Development was accomplished with either diaminobenzidine-based Elite (for Iba1) or Vector Red (other immunostains) kits (Vector Laboratories, Burlingame, CA).

### Data Analysis

#### Pre-processing and Coregistration

An elliptical 3D Gaussian apodization filter (sigma = 0.42) was applied to raw k-space data to improve SNR by down-weighting high-frequency signals and noise. Filtered k-space data were reconstructed to create magnitude and phase images using custom software written in Python[33] scripting language. Magnitude images were coregistered using a multi-step process. Briefly, images from each animal were skull-stripped with FSL’s BET[34] and then linearly registered to a single high-quality reference data set using FSL’s FLIRT[34]. A population average image was created and all data sets were linearly registered to the population average. Final registration to the population average was achieved via FSL’s nonlinear registration tool, FNIRT[34], with the affine matrix from the second linear registration step as a starting estimate.

#### Corrections and Parametric Mapping

All skull-stripped, coregistered images were corrected for inter-day variation in receiver gain settings by linearly scaling histogram intensities—while preserving native contrast—to match the reference images used in the initial coregistration step. The magnetization transfer ratio (MTR) was calculated as:

$$MTR\;=\;\frac{M_{0}-M_{sat}}{M_{0}}$$ (1)

where M_sat_ and M_0_ are magnetization values obtained from 3D GRE sequences with and without the saturation RF pulse, respectively (see MRI above). MTR maps were calculated for all animals and timepoints individually, and then pooled according to timepoint (Baseline, Week 6, Week 6+6) to create population averages. All corrections and parametric estimations were performed with software written in-house.

#### Statistical Analysis

Non-parametric permutation-based analyses were performed using FSL’s RANDOMIZE tool employing Threshold-Free Cluster Enhancement[35] with corrections for repeated measures consistent with our longitudinal design. No assumptions were made regarding distribution of means or variances. Paired and un-paired non-parametric t-tests compared voxel-wise MTR values in cuprizone-treated mice between Baseline and Week 6, Baseline and Recovery, and Week 6 and Recovery to identify significant changes at each time point.

#### Gene Expression

The Allen Institute for Brain Sciences Anatomic Gene Expression Atlas (AGEA)[36,37] was used to assess relative genetic homogeneity within the CC. “Seed” voxels were chosen at multiple points within the CC along the rostrocaudal and mediolateral axes. Both correlation and cluster maps for each seed voxel were visually inspected. The Gene Finder utility was then used to identify genes of interest (those with highest correlation or highest fold change compared to other structures) in our seeds in the CC. In situ hybridizaton (ISH) slides for the returned genes were then manually reviewed to ensure accuracy. Candidate genes were identified based on a visual inspection of anatomical distribution of gene expression energy. Three-dimensional renders of candidate gene expression energy and density[36,37] throughout the C57BL/5 P56 male mouse brain were then visualized using the Allen Institute’s Brain Explorer 2 desktop application. Expression energy distribution was qualitatively compared to patterns of demyelination observed in our 3D MTR maps.

## Results

### Reduced weight gain is associated with cuprizone diet

**Fig 1:**
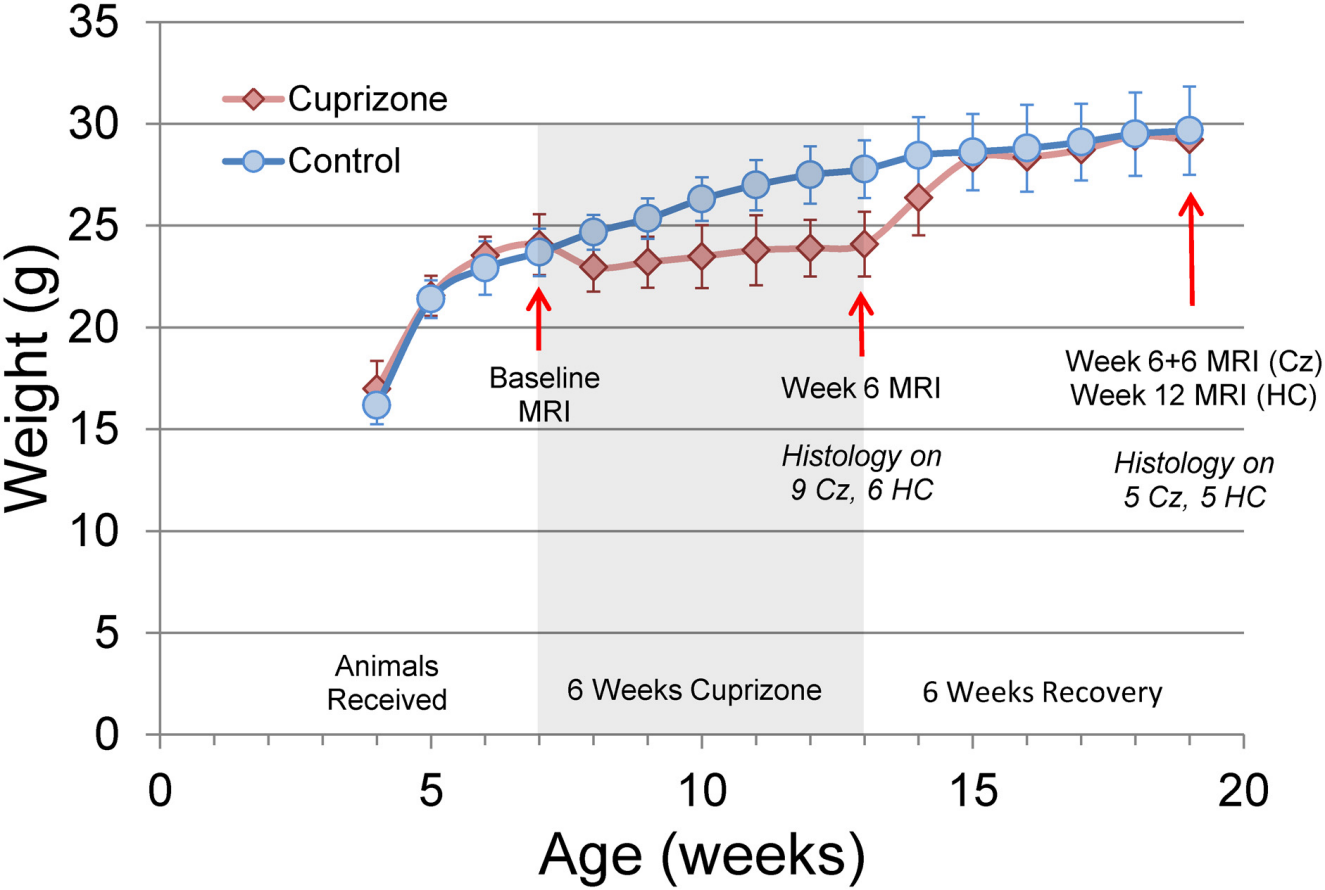
Weight Gain and Study Design. Average weight of animals over the course of the study (error bars represent one standard deviation). Cuprizone diet was administered for 6 weeks (shaded area) beginning 3 weeks after delivery. After one week on cuprizone the treatment group (Cz) exhibited lower weight than healthy controls (HC; *p<0.005); weight gain increased one week after cuprizone was removed from the diet, and two weeks into recovery no difference can be seen between the two groups’ weights.

Animals lost approximately 5% of their body weight during the first week of cuprizone feeding (p<0.005) followed by gradual weight gain over the next 5 weeks, consistent with previous observations.[11,38–40] Non-cuprizone fed mice gained weight roughly 3 times faster (0.65g/wk vs 0.23g/wk) than cuprizone-fed mice during treatment, and group weight averages were significantly different until one week after the treatment group returned to normal chow as shown in Fig 1.

### Longitudinal ROI analysis of MTR demonstrates regional heterogeneity in demyelination and remyelination

**Fig 2:**
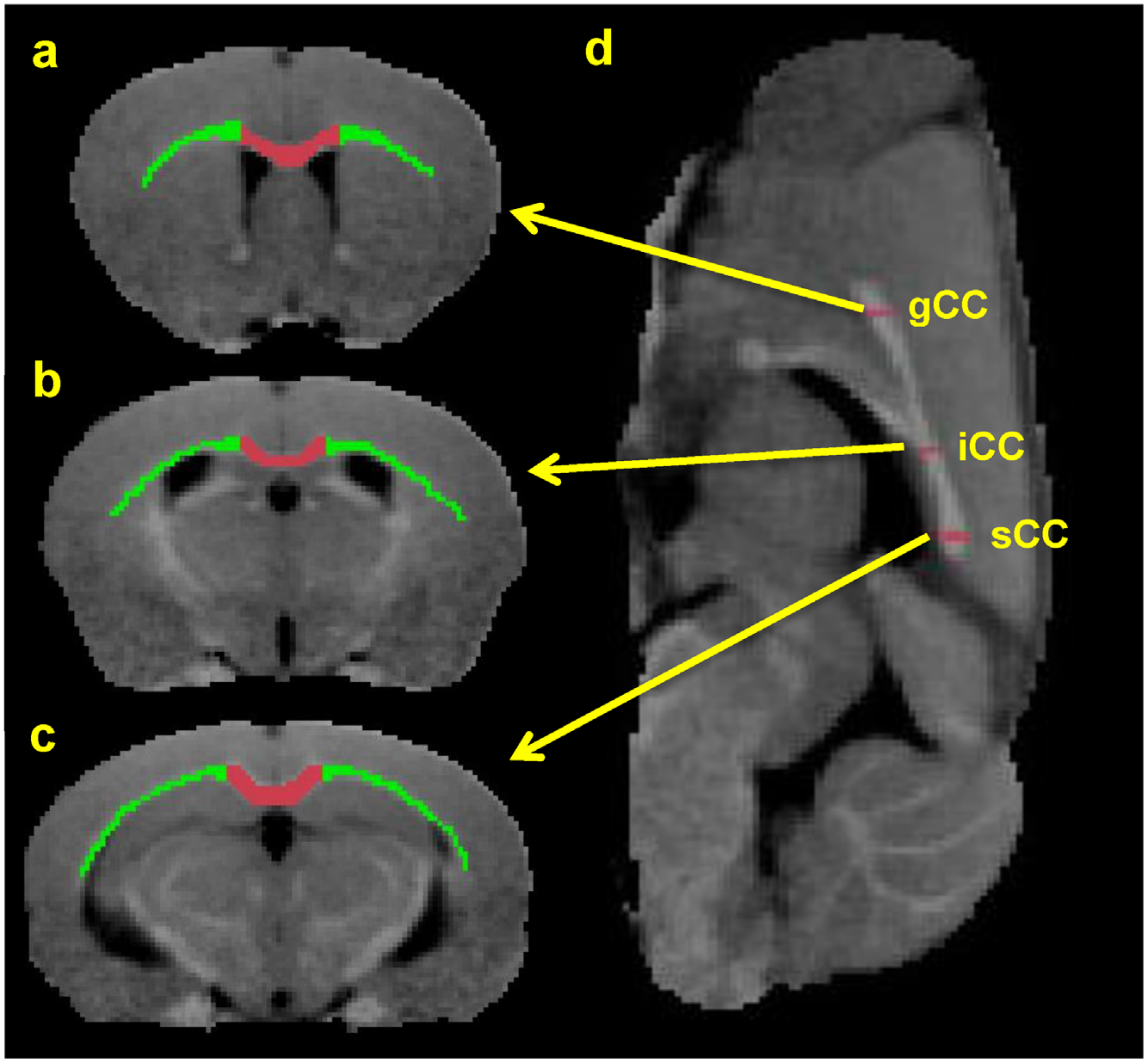
ROI Selection on Coronal MR Images. Colored ROIs used in scatter plots below are overlaid on population averaged baseline MTR images. Medial CC is red, lateral CC (including EC) is colored in green on coronal images. A sagittal view with arrows corresponding to the locations of the slices shown in (a-c), corresponding to genu (gCC), body/isthmus (iCC), and splenium (sCC), respectively, is shown in panel (d).

**Fig 3:**
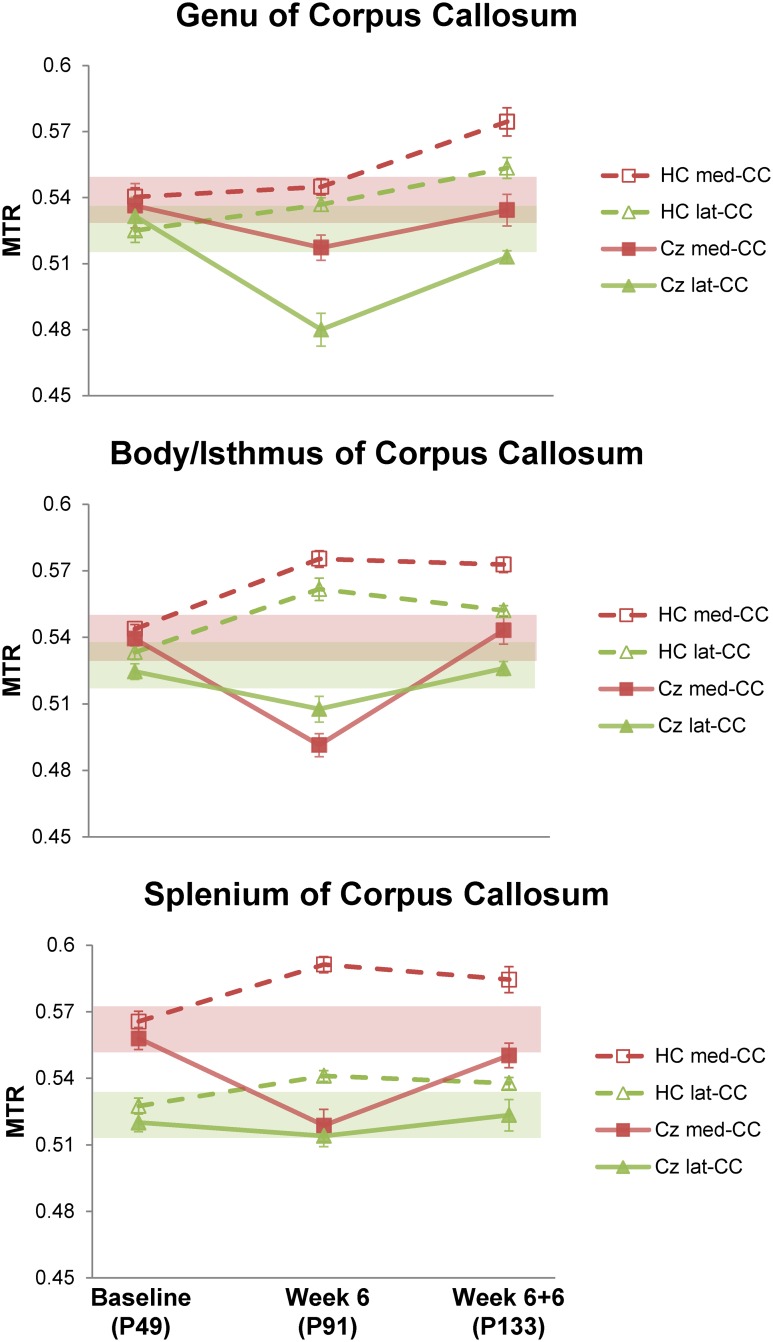
Longitudinal ROI-average MTR Compared to Healthy Age-Matched Controls. ROI-average MTR scatter plots for the medial- and lateral-CC ROIs illustrated in Fig 2(a)–2(c). Values represent population average within an ROI. Dashed lines/hollow markers represent data from healthy age-matched controls (n = 10, 4, 5, for Baseline (age postnatal (P) 49), Week 6 (P91), Week 6+6 (P133), respectively); data from cuprizone treated animals are shown with solid lines/filled markers (n = 14, 14, 5, for Baseline, Week 6, Week 6+6, respectively). Error bars represent mean standard error for each group. The shaded bands illustrate the 95% confidence interval for baseline values (±0.01).

We pooled callosal structures into two categories for ROI analysis: medial and lateral corpus callosum, referred to here as med-CC and lat-CC, respectively. Caudal med-CC likely includes adjacent structures such as fornix, dorsal hippocampal commissure, and cingulum. Fig 2 shows the distinction used here for lateral and medial corpus callosum. Three coronal slices were selected representing genu of CC (gCC, Bregma +1mm), isthmus of CC (iCC, Bregma -1mm), and splenium of CC (sCC, Bregma -2mm). Briefly, the med-CC corresponds to the center-most region demarcated by the apex of the lateral arches, consistent with similar studies; lat-CC thus includes all MRI-visible white matter distal to the apex of the lateral arches. We adopted this approach for two primary reasons: (1) various atlases disagree on location and extent of EC; (2) while distinguishable on histology, MRI partial volume effects limit differentiation of CC from small adjacent white matter structures. Longitudinal changes in population-average MTR values are demonstrated in Fig 3 for all cuprizone mice (solid lines) and healthy controls (dashed lines) at Baseline, Week 6, and Week 6+6.

Substantial reductions in MTR were seen along the entire length of the CC midline after 6 weeks of cuprizone (iCC p < 0.5x10^-6^, sCC p = 0.001), with an increase in absolute change following a rostro-caudal gradient. Lateral CC MTR decrease was significant in the genu (p = 0.0005) and isthmus (p < 0.05). Importantly, a striking rostro-caudal gradient of decreasing effect is observed in the lat-CC—opposite of med-CC. Indeed, only a small change was seen in lat-CC at the level of the isthmus, and caudal lat-CC appears essentially preserved. MTR returned to near-baseline values in all regions except lateral gCC (p < 0.05) after 6 weeks recovery on normal chow (Cz Week 6+6). MTR values at Cz Week 6+6 generally hover right between those obtained at Baseline and Week 6, suggesting gradual return to some level of contrast at Week 6+6 that resembles Baseline.

MTR in all structures in control mice matched well with those of the cuprizone group at baseline. However, by Week 6, MTR in control mice increased in all ROIs compared to baseline, and generally continued to increase throughout the 12 week study. Fig 3 compares MTR changes in controls versus cuprizone mice at Baseline, Week 6, and Week 6+6 (recovery).

### MTR parametric maps reveal complex pattern of demyelination across whole brain

**Fig 4:**
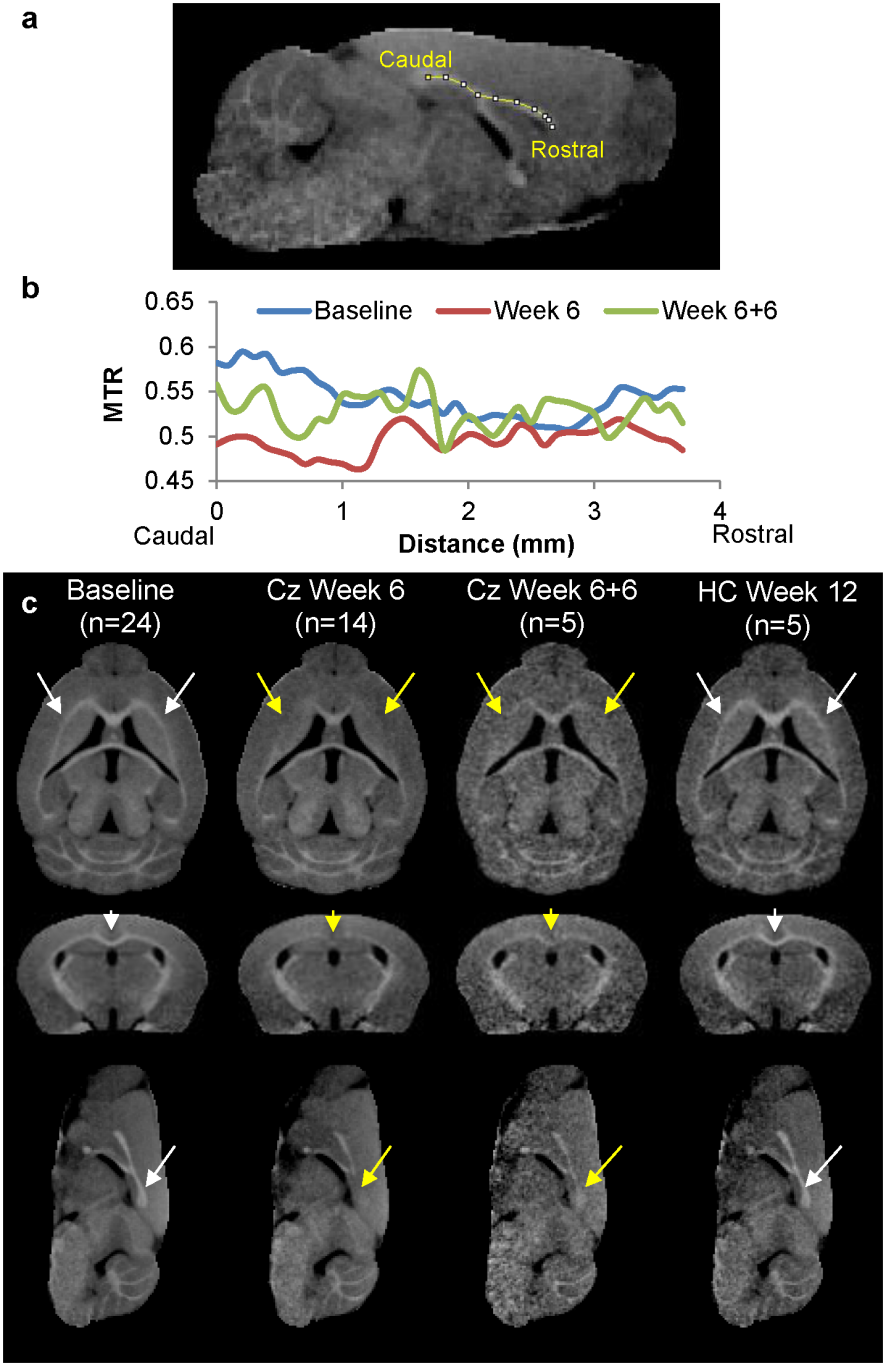
Population Average Orthogonal MTR Parametric Maps. (a,b) Population average MTR projection along midsagittal CC from Caudal (splenium) to Rostral (genu) at Baseline (blue), Week 6 (red), and Week 6+6 (green). (c) Population-average MTR maps at baseline, Cz Week 6, Cz Week 6+6, and HC Week 12. Note that SNR increases with the number of images averaged, resulting in poorer image quality in Cz Week 6+6 and HC Week 12 images.

**Fig 5:**
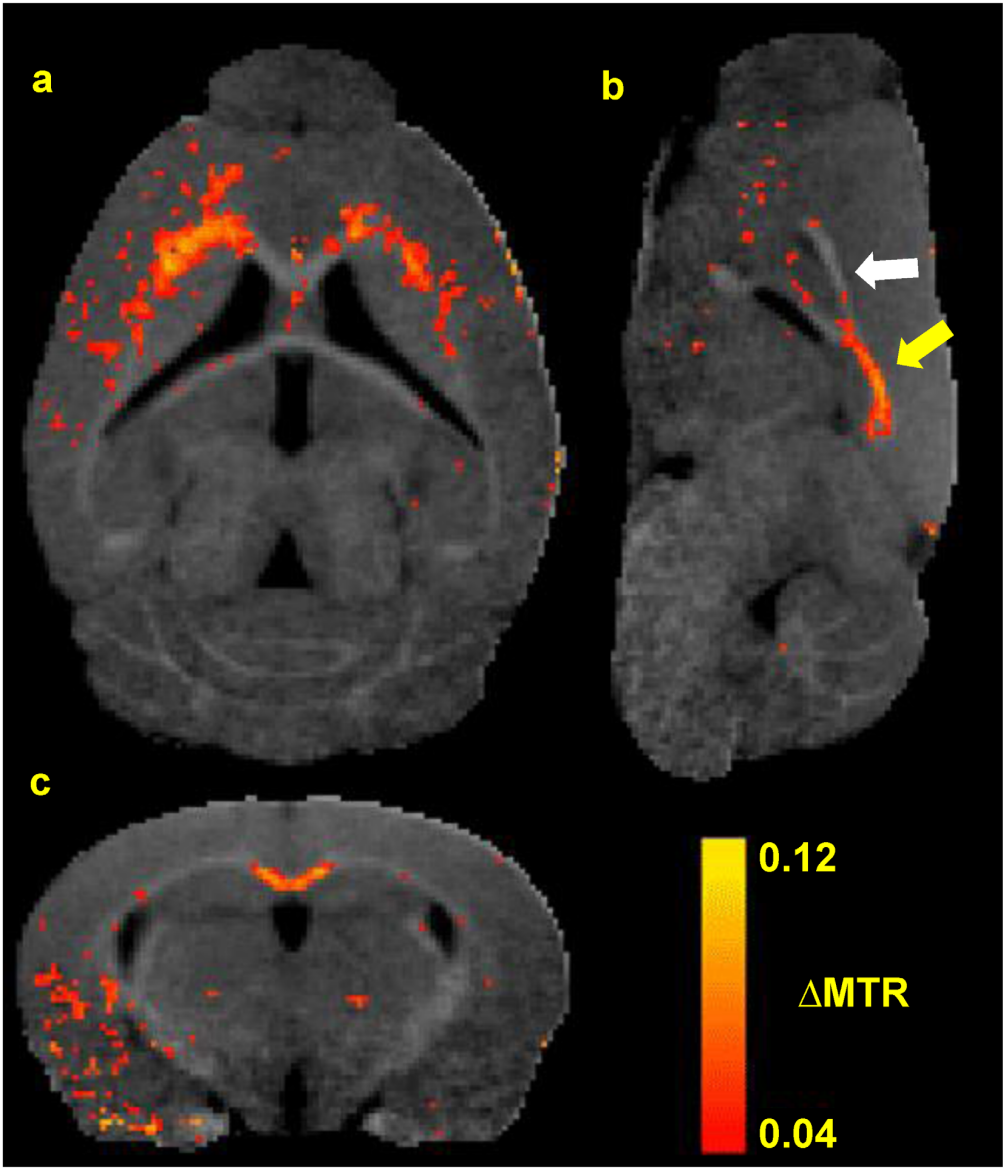
Population Average Week 6 ΔMTR Parametric Maps. Horizontal (a), sagittal (b), and coronal (c) views of the mouse brain. Color overlay represents areas of MTR decrease (paired t-test, p<0.01) after 6 weeks of cuprizone as compared to baseline. The caudal-rostral pattern of demyelination along the midline is illustrated in panel b (arrows).

**Fig 6:**
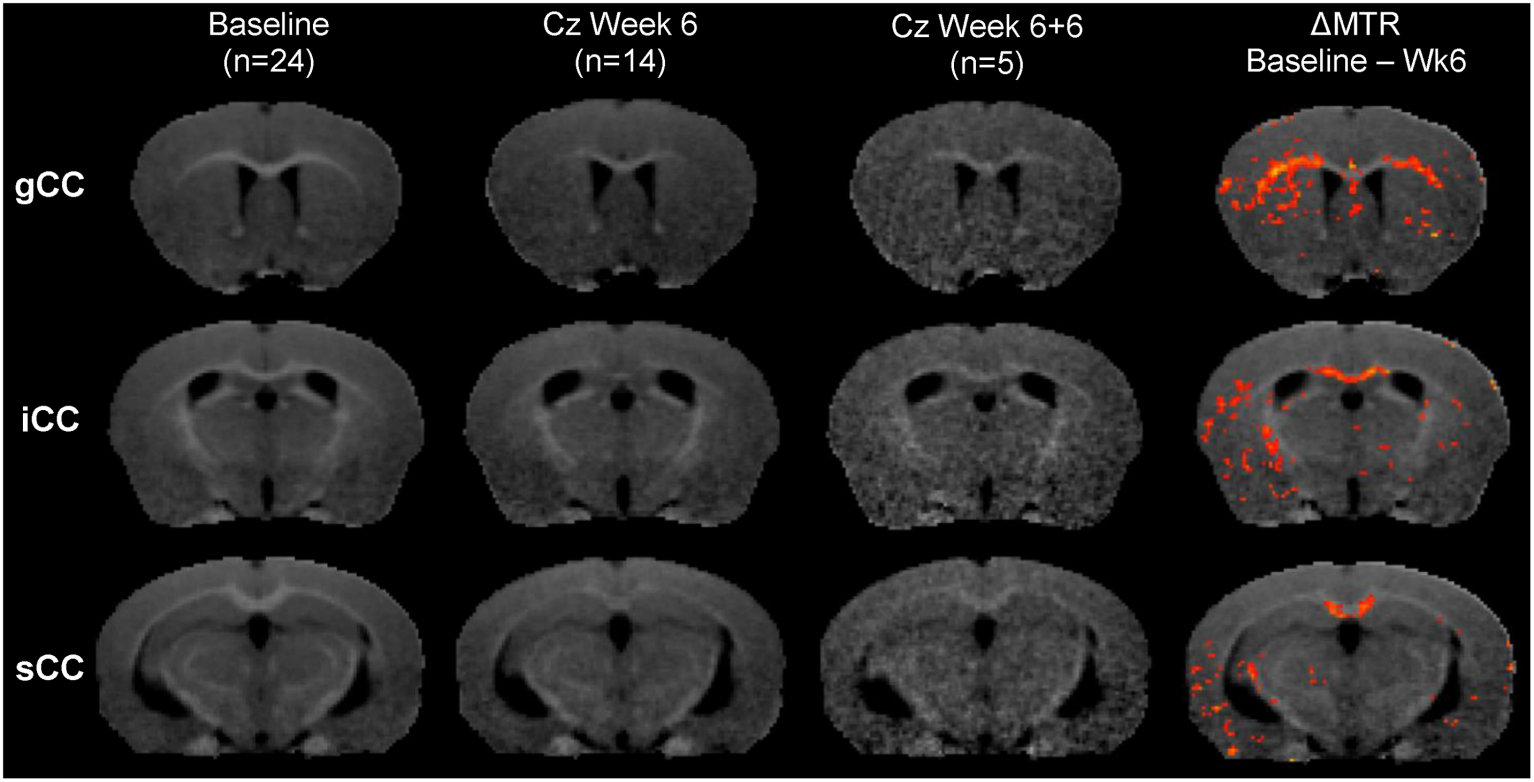
Population averaged Coronal MTR maps. MTR maps at the level of gCC (top row), iCC (middle row), and sCC (bottom row). ΔMTR (p < 0.01) values are overlaid in false color (see Fig 5 for color bar) in the far right column. Note reduced image quality at Cz Week 6+6 due small number of images included in the average (n = 5) compared to Baseline (n = 24) and Cz Week 6 (n = 14).

Population average MTR maps for Baseline, Cz Week 6, Cz Week 6+6 (cuprizone recovery) and Week 12 (control) are compared in Fig 4. Baseline images for one control animal were discarded due to incomplete image acquisition. An MTR intensity projection along the midsagittal CC (Fig 4a and 4b) shows natural variance in MTR along the CC in healthy animals at baseline (blue line). This caudal-rostral gradient is entirely eliminated after 6 weeks cuprizone (red line), and only partially recovers after 6 weeks of normal chow post-cuprizone (green line). At Cz Week 6, rostral lat-CC/EC and caudal med-CC have become isointense with surrounding gray matter, suggestive of distinct demyelination (Fig 4c, yellow arrows). Limited corpus callosum contrast returns at Cz Week 6+6, but remains hypointense compared to control (white arrows). Fig 5 shows the population average baseline MTR map with the statistically significant (paired t-test p < 0.01) average ΔMTR (= MTR_Baseline_−MTR_Week6_) values overlaid in color. Panel (a) shows a horizontal slice demonstrating decreased MTR in the rostral lat-CC. A midsagittal view is shown in panel (b); the caudal-rostral pattern of decreased MTR in the medial CC is evident (yellow arrow: caudal demyelination; white arrow: rostral myelin preserved). A coronal slice (approximately bregma -1mm) is shown in panel c; here the medial CC displays decreased MTR whereas the lateral CC was unaffected. MTR at Cz Week 6+6 was not significantly different from either Cz Week 6 or Baseline, suggesting substantial yet incomplete recovery. Consistent with previous reports, ventricular enlargement was observed at Week 6 but was essentially resolved after 6 weeks of recovery.[1,12,25,41] MTR depressions scattered throughout the ventral cerebral cortex may be artifactual and can be attributed to SNR decreasing with distance from the surface receive coil. Decreased MTR was observed in the CBLL peduncles and limited diffuse decreases throughout deep gray matter and cortex were seen in parametric maps (p < 0.05; not shown). Coronal MTR maps corresponding to gCC, iCC and sCC in Fig 6 illustrate MTR contrast at Baseline, Cz Week 6, and Cz Week 6+6.

### Quantitative histological analyses correlate well with MTR

**Fig 7:**
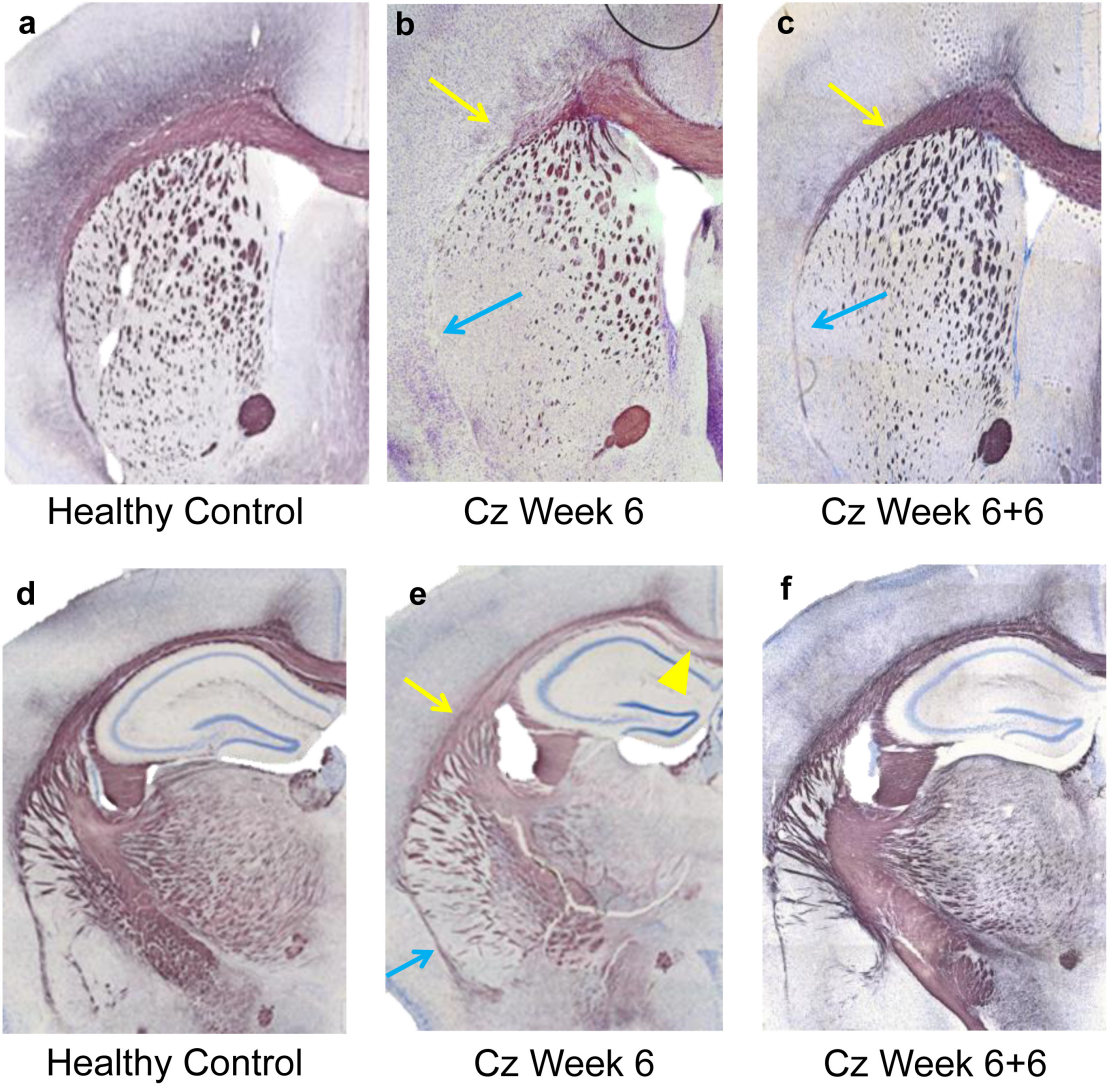
Black Gold II Stain for Myelin in Caudal and Rostral CC. BGII stains roughly corresponding to gCC (a-c) and sCC (d-f) ROIs in Fig 2. Age-matched control with normal myelinated lat-CC/EC (a); substantial demyelination in rostral lat-CC/EC (arrows) after 6 weeks Cz (b); lat-CC/EC partially recovered after 6 weeks on regular chow (c). Reduction in myelinated fibers in caudoputamen is observed both at Week 6 and Week 6+6 compared to control. Stark demyelination is evident in the caudal med-CC (e, arrowhead), while lat-CC/EC (arrow) is only mildly demyelinated after 6 weeks cuprizone (e) compared to control (d). Remyelination in lat-CC/EC and med-CC is substantial yet incomplete after 6 weeks recovery (f). Blue arrows indicate demyelination in EC beyond the extents of MRI-visible lat-CC/EC.

**Fig 8:**
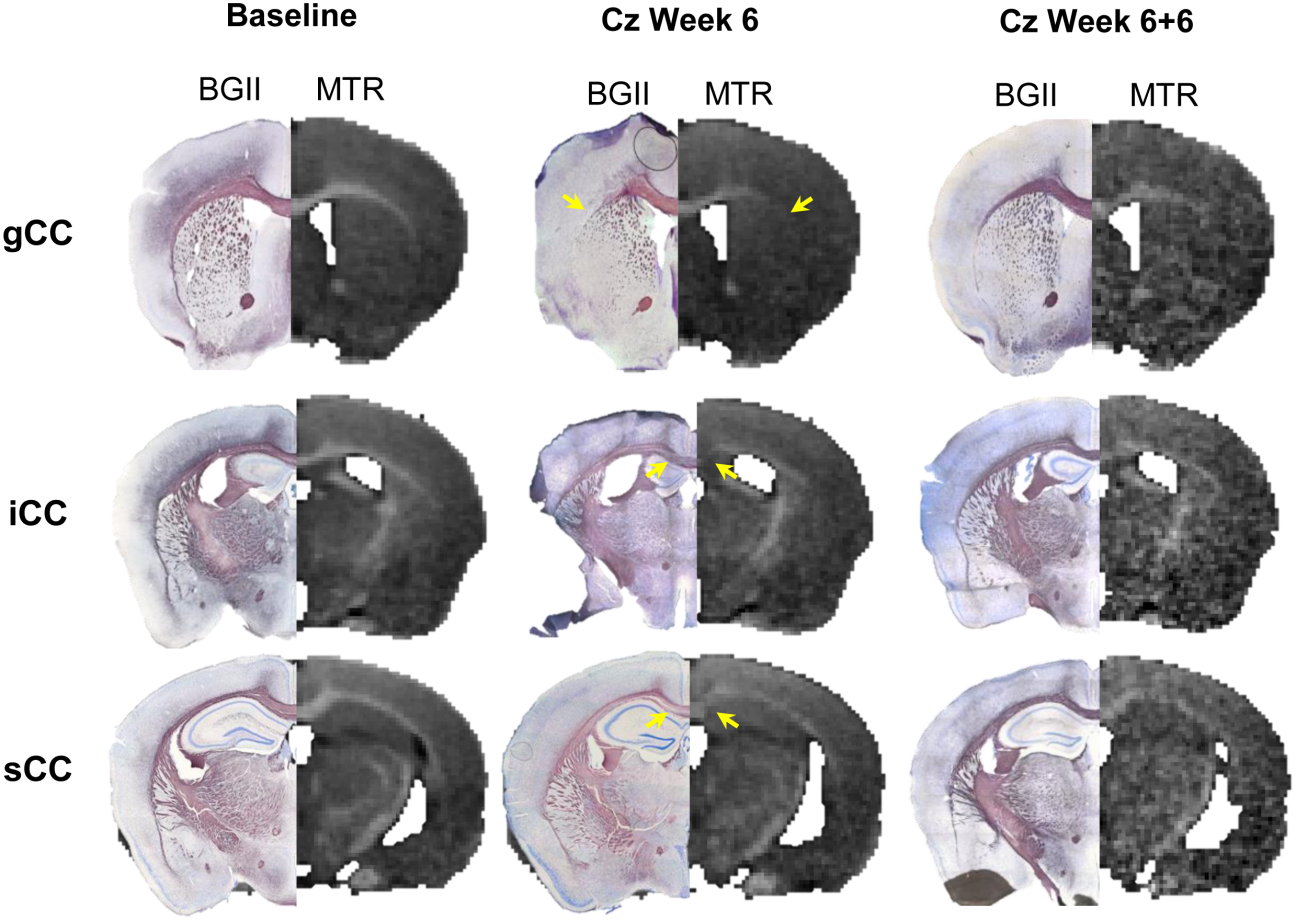
BGII/MTR Hybrid images. BGII sections compared to MTR maps in gCC, iCC, and sCC. Hybrid BGII/MTR images demonstrate MTR contrast and corresponding BGII staining at the level of gCC (first row), iCC (second row), and sCC (third row) at Baseline (left), Cz Week 6 (middle.), and Cz Week 6+6 (right). Loss of MTR contrast at Week 6 matches changes in BGII staining, representing demyelination (yellow arrows). The black circle in the cortex above gCC at Cz Week 6 is a coverslip bubble and should be ignored.

Quantitative analyses of tissue samples with BGII stain for myelin are summarized in Table 1. Any part of lat-CC/EC that was not MRI-visible, and thus not included in the green ROIs shown in Fig 2, was excluded from this analysis. Demyelination is evident and variable along the length of the CC. Both MTR and ΔMTR average ROI values correlated well with the percentage of demyelinated area; Pearson’s correlation coefficients (r) are shown in Table 2 along with linear regression parameters. Overall, ΔMTR was more highly correlated with percent demyelinated area than was MTR. Sections obtained at bregma +1mm stained with BGII shown in Fig 7 illustrate myelination in the lat-CC in a healthy control (a), after 6 weeks cuprizone challenge (b), and 6 weeks cuprizone + 6 weeks recovery (c). Complete demyelination is evident in the rostral lat-CC after 6 weeks of cuprizone, yet med-CC myelin is preserved. Comparing panels a and c it is evident that remyelination in the lat-CC is incomplete after 6 weeks recovery following 6 weeks of cuprizone. Panels d-f demonstrate patterns of myelination at bregma -1.5mm in healthy control (d), after 6 weeks cuprizone (e), and after 6 weeks recovery post-cuprizone (f). Caudal med-CC is heavily demyelinated (arrowhead, e), and lat-CC/EC show moderate demyelination compared to control (d). All structures show dense stain for myelin after 6 weeks of recovery (f). Hybrid images created from BGII and MTR images in Fig 8 demonstrate that MTR contrast reflects BGII staining in gCC, iCC, and sCC at Baseline, Cz Week 6, and Cz Week 6+6. Limits of MRI-visible EC compared to the full extent of the structure are clearly evident.

**Table 1:** BGII stain for myelin in corpus callosum—demyelination as percent area.

		% BGII Demyelination (SD)		
Group	Section	Whole CC	Medial CC	Lateral CC
**Cz Week 6**	gCC	9.7 (±0.6)	2.3 (±1.9)	17.2 (±3.6)
	iCC	8.2 (±0.9)	20.7 (±4.8)	3.4 (±0.8)
	sCC	16.3 (±2.6)	30.7 (±8.5)	8.9 (±3.0)
**Cz Week 6+6**	gCC	2.6 (±0.3)	0.9 (±0.4)	4.0 (±0.6)
	iCC	4.9 (±0.9)	6.5 (±2.4)	4.3 (±0.9)
	sCC	2.3 (±1.0)	3.6 (±2.1)	1.8 (±1.0)

**Table 2:** BGII—MTR linear regression.

Region	BGII vs MTR			BGII vs ΔMTR		
	*Equation*	*r*	*p*	*Equation*	*r*	*p*
Whole CC	y = -1.7x + 0.1	-0.55	0.26	y = 2.1x + 0.04	0.63	0.18
Medial CC	y = -3.3x + 1.8	-0.58	0.23	y = 4.8x + 0.02	0.82	<0.05
Lateral CC	y = -2.4x + 1.3	-0.87	<0.05	y = 2.4x + 0.03	0.83	<0.05

Pearson’s correlation coefficients (r) demonstrate BGII % demyelination is negatively correlated with MTR, and positively correlated with ΔMTR. Linear regression parameters: *y* represents BGII % demyelination; *x* is either MTR % or ΔMTR (%).

### T_2_-weighted images provide qualitative and semi-quantitative estimates of myelin content

**Fig 9:**
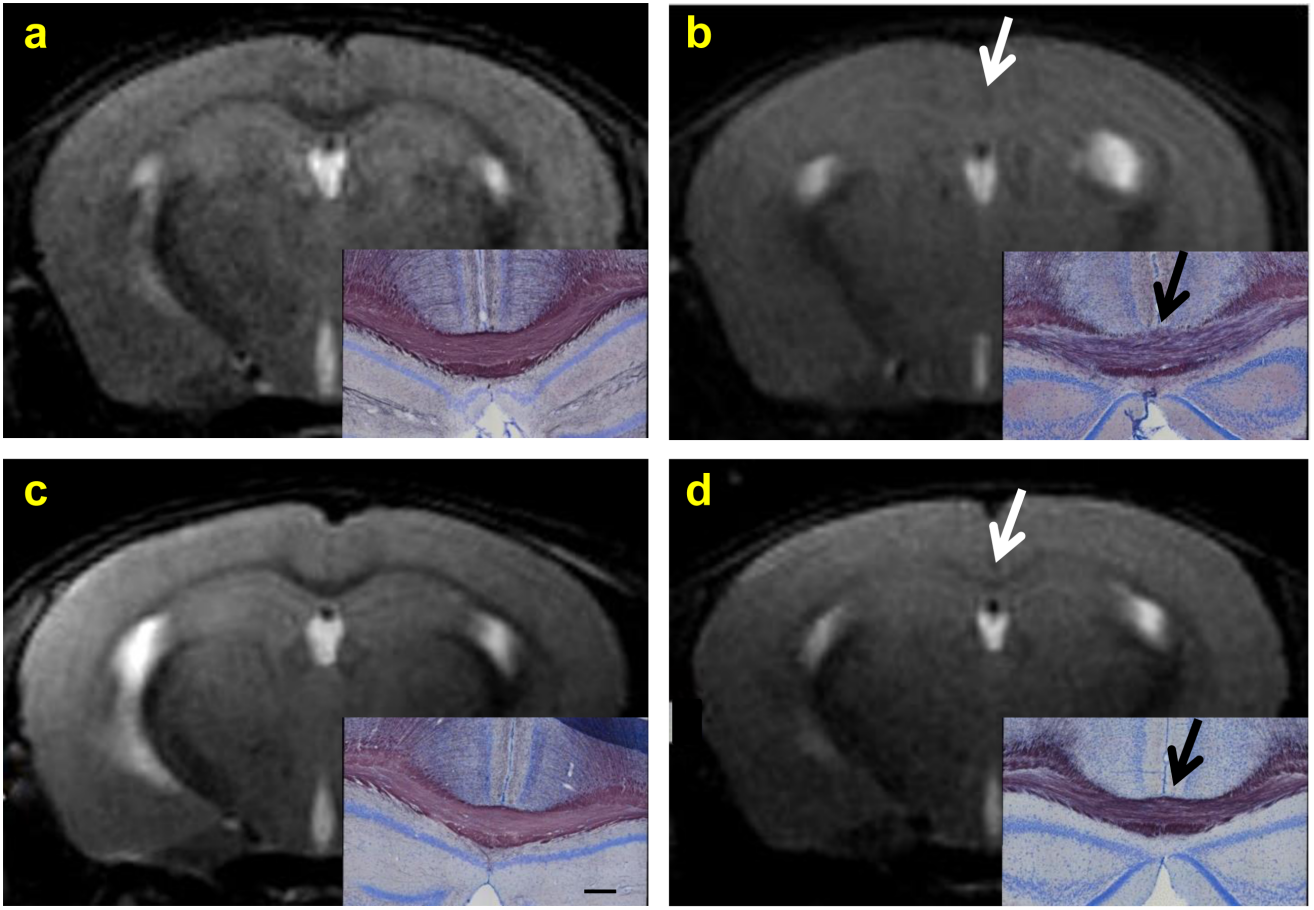
T_2_-w MRI Compared to BGII. T_2_-w RARE coronal images and pathologic sections stained with Black Gold II for myelin (insets, scale bar 200μm) from mice fed normal chow (a and c), after 6 weeks on cuprizone chow (b) and 6 weeks after stopping cuprizone chow (d). MRI from age-matched control mice (a and c) show low signal intensity in the corpus callosum and corresponding normal myelin on histology. After 6 weeks of cuprizone exposure (b), the corpus callosum has increased signal intensity (now isointense with cortex) and corresponding decreased staining for myelin on histology. Six weeks after being taken off of cuprizone, MRI shows return of hypointensity in the corpus callosum and corresponding increased staining for myelin on histology (d).

T_2_-weighted (T_2_-w) images give excellent GM/WM contrast in-vivo. In age-matched healthy controls, the med-CC and lat-CC both are clearly hypointense compared to GM as shown in Fig 9a and 9c. After 6 weeks of cuprizone treatment the GM/WM contrast is eliminated as callosal regions are isointense with GM, consistent with loss of myelin as shown via histology in Fig 9b inset. Remyelination is indicated by increased GM/WM contrast in T_2_-w images (Fig 9d) 6 weeks after removal of cuprizone. Black Gold II stain for myelin is fairly homogenous in the CC in healthy controls (panels a,c insets) compared to the disorganized and inhomogeneous appearance in demyelinated CC (panel b inset). Myelination appears somewhat more organized and homogenous after 6 weeks of recovery (panel d inset), indicating partial, yet incomplete remyelination.

**Fig 10:**
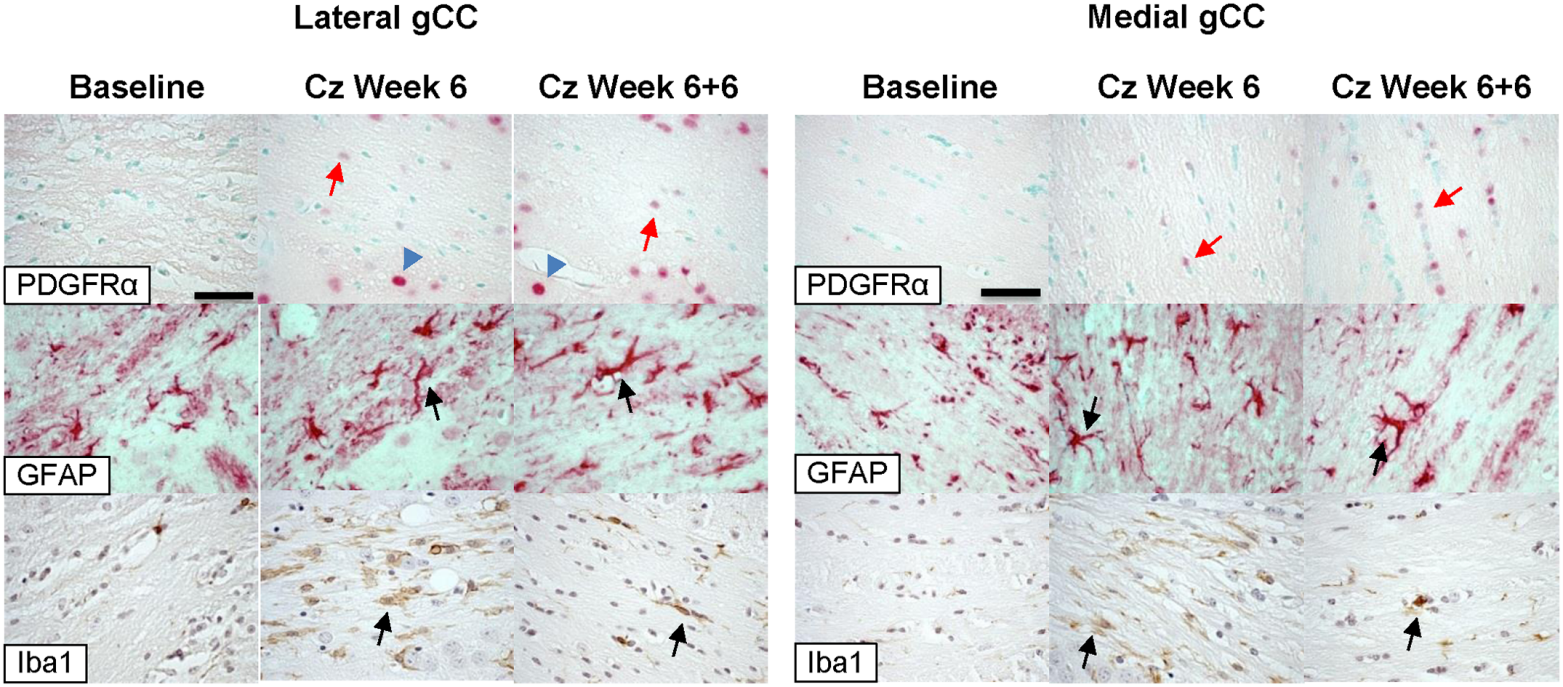
Immunohistochemical analyses of gCC. OPCs stained with PDFRα appear red (red arrows), often in a tight line with other cells, in the top row (25μm scale bar applies to all images). Note that neurons (blue arrowheads) expressing PDGFRα exhibit large, round nuclei. Activated astrocytes, stained red, are identified by an enlarged cell body and stalky processes in the second row (arrows). Microglia appear brown in the bottom row (arrows).

We performed additional immunohistochemical analysis of baseline, Cz Week 6, and Cz Week 6+6 mice using antibodies to glial fibrillary acid protein (GFAP, an astrocyte marker), microglia (Iba1), and PDGFα receptor (a marker of proliferating oligodendroglial precursors). Microglia were markedly increased in affected areas of the corpus callosum after cuprizone treatment compared to baseline animals, with incomplete normalization of microglial signal after 6 weeks recovery. Microglial burden at Cz Week 6 corresponded approximately to the degree of demyelination at various sites: greater microglial burden in lateral gCC than medial gCC, and less microglial burden in lateral iCC and sCC than in medial iCC and sCC, respectively. Oligodendroglial proliferation, as assessed by PDGFα receptor expression, was modestly increased after cuprizone treatment, and more strikingly so in the recovery phase, throughout all affected areas of the corpus callosum. Fig 10 shows representative high-magnification images of medial and lateral gCC stained for GFAP, PDGFRα, and Iba1, at Baseline, Cz Week 6, and Cz Week 6+6.

### Gene expression in the CC is heterogenous and aligns with patterns of demyelination and MTR depression

Distinct zones of unique gene expression are evident within the CC, exhibiting caudo-rostral and medio-lateral gradients of regional gene expression correlation. Example seed positions in AGEA: 7.113, 1.873, 5.620 for caudal medial CC; 4.477, 3.302, 5.620 for rostral medial CC. Myelin basic protein (MBP) in-situ hybridization (ISH) expression energy (experiments 112202838 and 79632288)[36,37,42] exhibits the opposite rostro-caudal and medio-lateral patterns of decreased MTR—and demyelination—observed after 6 weeks of cuprizone exposure. That is to say, less demyelination and MTR decrease was observed in the CC where MBP expression is greatest, demonstrating an inverse correlation between MBP mRNA expression and demyelination/MTR decrease.

## Discussion

The primary findings of this study were: (i) acute cuprizone-induced demyelination is region-specific and varies along both rostrocaudal and mediolateral gradients; and, (ii) histological estimates of myelin content correlated well with MTR values obtained in-vivo in this model. We further showed that MTR increases in healthy mice throughout adolescence and adulthood, which may indicate continued myelin development and maturation, demonstrating the need for appropriate age-matched controls in these studies.

Cuprizone induced demyelination showed marked spatial heterogeneity with strong caudal-to-rostral and medial-to-lateral patterns. The caudal-to-rostral pattern of cuprizone-induced demyelination in the medial corpus callosum has previously been described.[11,16,23,38] Less known is the medial-to-lateral demyelination pattern that we observed in conjunction with the caudal-to-rostral pattern. We are among the first to report in-vivo 3D MTR mapping in the cuprizone mouse model and here present the highest resolution 3D characterization of spatial patterns of cuprizone-induced demyelination in medial and lateral CC to date.

Histological estimates of myelin content correlated well with MTR values (r = -0.58 and -0.87 in medial and lateral CC, respectively) obtained in-vivo. While MTR changes associated with demyelination were modest (~5–10%) compared to histological assessment of myelin loss (up to 30%), ΔMTR was more highly correlated with demyelination throughout the CC (r = 0.82 and 0.83 in medial and lateral CC, respectively).

It is evident that the cuprizone-induced injury-repair dynamic is complex and highly varied across the brain. Perhaps varying genetic niches within the corpus callosum—and likely in other regions in the central nervous sytem—contribute to the region-specific nature of demyelination in this model. Gene expression is heterogeneous throughout the CC, and patterns of demyelination may coincide with patterns of basal gene expression. This regional heterogeneity may be essential in not only understanding the mechanisms involved in this model, but also in appropriately and effectively applying it to study human disease.

### MTR is a sensitive, but not specific, measure of demyelination in-vivo

MTR is sensitive to demyelination but is not specific: MTR values are substantially changed—typically decreased—by demyelination, but also by inflammation and edema.[19,22] MTR has been shown to be highly positively correlated with axonal density, perhaps even more so than with myelin content;[43] however, little or no axonopathy is observed in this model. Because the cuprizone model does not induce substantial edema or axonopathy, we can be confident that the changes observed in MTR here are reflective more of demyelination than any other pathology. Preserved axonal density combined with increased astrocyte infiltration and activated microglia (Fig 10) will contribute to MTR values and likely obscure MTR decreases due to demyelination alone. So, ΔMTR may be viewed as a measure of injury outpacing repair and development in this model. Histological staining with Black Gold II (Fig 7) and subsequent quantification (Table 1) match well with patterns of MTR changes shown in Figs 3–6, thus validating MTR as an effective measure of demyelination in this model. In addition to the corpus callosum, MTR decreases in cerebellum, thalamus, cerebral cortex have been reported;[12] we observed similar patterns but did not focus on these brain regions in this analysis. Zaaouri, et al, used thalamus as a reference tissue for normalization and reported[13] lower MTR values in the CC (0.35–0.25 compared to 0.55–0.45) than in the present work. However, the thalamus is not a suitable reference region as demyelination and reduced MTR after 6 weeks of cuprizone treatment have been demonstrated in this area.[12] Thiessen, et al,[20] reported MTR at 7T in rostral CC with slightly lower baseline (0.5) and greater depression at Week 6 (0.38) than what is shown in this paper. Fjær, et al,[12] performed a similar longitudinal MTR study using a 7T MRI instrument and MT imaging parameters similar to ours, and report ΔMTR that are in excellent agreement with our findings. The correlation we observed between MTR and histologically-determined myelin content is consistent with the work of Fjær and others.[13] The magnitude of response to demyelination is substantially lower in MTR compared to histology, but is nevertheless clear and significant. The attenuation in MTR effect size may be due to MTR’s sensitivity to axonal density and infiltration of astrocytes, microglia and macrophages, which may increase observed MTR and confound the measurement.

### Acute demyelination is region-specific and expresses heterogeneous pathology

Estimates of percentage of myelinated axons present in the adult CC vary greatly due to inconsistencies in definitions of ‘mature,’ and application of minimum axonal diameter thresholds. Early studies reported only roughly 30% of mature axons in CC are myelinated;[44] conversely, more recent studies indicate that 80–90% of axons are myelinated in the healthy mouse CC.[16,17,45] Our method of quantifying myelination assesses the area of tissue that stains for myelin compared to total area of interest. Using this technique we report baseline as 100% myelination and then demyelination is reported as a fractional area in the CC that does not stain for myelin.

Qualitatively, microglial burden tracked with severity of demyelination and MTR reductions. Microglial infiltration was observed in all CC ROIs, with higher burden in areas exhibiting greatest demyelination. Reactive astrocytes were identified by their GFAP-positive cytoplasmic processes. This morphologic feature of reactive astrocytes is more difficult to assess quantitatively and is further complicated by subtle variations in astrocyte morphology in the relatively loose lateral corpus callosum compared to the more dense medial tissue. Additionally, given the regional differences in astrocyte morphology, making comparisons between lateral and medial CC astrocyte infiltration is challenging. Quantitative analysis of PDGFα receptor stains can also be ambiguous due to (1) non-specific staining of OPCs and neurons, (2) often only a small number of positive oligodendroglia were observed, so sampling variation can lead to gross under- or overestimation of effect, and, (3) the staining of oligodendroglia, in contrast to neurons, was quite variable and sometimes light in these sections. This may be due to the fact that as oligodendrocytes mature they lose expression of this antigen, thus reflecting biological phenomenon rather than a technical problem.

Axonal caliber varies along a rostrocaudal gradient in the human CC,[46,47] and may influence susceptibility to demyelination and capacity for future remyelination. While true in humans, ultrastructural studies have demonstrated that initial axon caliber does not vary significantly across the mouse CC,[16,45] and remains constant with age,[44] and thus does not predict demyelination in this model. However, a more thorough characterization of axon caliber across the whole CC as the brain ages will be necessary to adequately address this possibility.

Histopathological heterogeneity between the small white matter structures adjacent to CC has been observed at a variety of time points and illustrates the importance of distinguishing between structures such as CC and dorsal hippocampal commissure, among others.[11] This distinction is interesting histologically, but is challenging to detect using MRI. Despite having collected the highest resolution in-vivo MRI to date, our resolution was too low to reliably distinguish these structures. Thus, separate comparisons between histology and MRI for these structures were not performed. ROI analysis separating lat-CC and med-CC reveals a complex pattern of demyelination both histologically (Table 1) and via MRI (Figs 2–5**)**: rostral CC demyelinates laterally but is relatively unaffected medially; caudal CC demyelinates medially, but is preserved laterally. So, there is a clear caudal-to-rostral and medial-to-lateral pattern of demyelination within the CC. A very recent study demonstrated a rostro-to-caudal pattern of demyelination in the cortical grey matter similar to what is shown here in lateral CC, wherein demyelination occurs earlier and with greater severity in motor cortices (rostral, proximal to gCC) than in the somatosensory cortices (caudal, proximal to iCC).[48] This result supports our observation of a complex pattern of demyelinating gradients throughout the mouse brain. We stress the importance of obtaining parametric maps to reveal the intricate and subtle spatial patterns of demyelination that correspond to those seen on histology, as shown in Figs 7–8.

It has been suggested that the lateral and medial CC can be combined for analysis in the rostral CC;[19] our results establish that the distinction between medial CC and lateral CC/EC is critical in accurately evaluating extent of demyelination and remyelination. Loss of myelin in the rostral lateral CC/EC adjacent to unaffected med-CC consistent with our observations has been previously shown,[6,41] though little analysis has been provided. Clearly, pooling medial CC and lateral CC in ROI analysis obscures underlying pathological heterogeneity and neglects essential model characteristics.

We observed few OPCs in demyelinated CC ROIs after 6 weeks of cuprizone treatment, consistent with the modest remyelination attempts prior to removal of cuprizone. Accordingly, greater numbers of OPCs were observed in the recovering CC 6 weeks after cuprizone removal (Fig 10). Oligodendrocyte precursor migration and differentiation is determined by a complex system of long-range and short-range cues and growth factors expressed during development,[49–51] and the process of progenitor migration and fate in the adult brain, particularly in disease state, is still unclear. Neural progenitor cells (NPCs) reside primarily in the subventricular zone (SVZ) in adult brains, located along the lateral wall of the lateral ventricles, and are also found in the subgranular zone (SGZ), adjacent to dentate gyrus.[52,53] A number of studies have explored the roles of the SVZ and SGZ in responding to cuprizone-induced demyelination.[54–59] The results from these studies combined with the patterns of demyelination and remyelination observed in the present work suggest that the rostral med-CC is preferentially supported by progenitor cells from the SVZ via the rostral migration stream, while the SGZ responds to injury in the caudal med-CC. Additional histopatholigical studies achieving more complete brain coverage are needed to elucidate how the different populations of progenitor cells contribute to repair and regeneration in this model, particularly with respect to the lat-CC/EC.

### Cerebral white matter may continue to develop in healthy mice throughout adolescence and adulthood

Previous DTI studies of maturation in the mouse brain suggest development plateaus around 5–6 weeks after birth,[60] and maturation is spatially and temporally heterogeneous throughout development.[61] Other work has suggested that MTR maps in the healthy mouse brain do not change with age and that age-matched controls may be superfluous.[19] However, a very recent study demonstrated age-related changes in T_2_-w and diffusion kurtosis imaging in the medial sCC, body of CC, gCC, and cortex between eight and twenty weeks of age.[48] Our results also indicate that age-matched controls are in fact essential in this model, particularly for quantitative MRI studies. Fig 3 shows MTR increases after Baseline in both medial and lateral CC, suggesting ongoing development and maturation. Indeed, the Week 6 MTR effect size increased when values were compared to age-matched controls rather than Baseline (average Cohen’s d = 1.5 [range 0.5–3.3] compared to Baseline; d = 2.7 [range 1.7–5.2] compared to age-matched controls). Further, it is clear that even 6 weeks after cuprizone cessation, MTR remains abnormally low compared to healthy age-matched controls. These data suggest that recovery to Baseline MTR does not represent true normalization with respect to healthy age-matched controls.

### Regional differences in gene expression may contribute to site-specific vulnerability

Regional heterogeneity in resistance and vulnerability to demyelination in the cuprizone model almost certainly involves complicated interactions of numerous enzymes, proteins, cofactors, and signaling pathways. Heterogeneous gene expression in the mouse CC suggests genetically distinct zones within the CC that coincide with varying degrees of demyelination. It was initially surprising to us that MBP is most abundant in those parts of the CC/EC that are most resistant to cuprizone-induced demyelination. MBP associated with the myelin membrane is responsible for the multilamellar structure of myelin and is highly sensitive to metal ion concentrations. Cuprizone administration can create imbalances in copper and zinc ion concentration, which could subsequently destabilize the MBP-membrane association, reduce myelin compaction, and degrade myelin quality. Perhaps it is the regionally increased expression of MBP that is responsible for—or at least an important contributor to—resistance to cuprizone-induced demyelination. However, a complete proteomic analysis is beyond the scope of this paper. We recommend additional, more complete proteomic studies of the healthy CC and surrounding structures to thoroughly characterize the genetic environment that may affect demyelination and remyelination in neurodegenerative disorders.

### Regional heterogeneity may hold the key to understanding the model

Compelling evidence has been given to suggest that the primary action of cuprizone is metabolic disruption, which leads to oligodendrocyte death. Our findings suggest a more complex story. Regional heterogeneity in demyelination and remyelination suggests distinctly varying microenvironments exist in the CC. Perhaps it is the varying spatial proximity to various NPC niches[49,50,52–59] coupled with heterogeneous expression of MBP RNA that determines, at least partially, the regional and site-specific nature of demyelination and remyelination in the cuprizone mouse model. A better understanding of spatial and temporal pathological patterns in the model is essential to determining how and why damage and repair occur. Thus, further study employing imaging techniques that are sensitive to demyelination and are capable of whole-brain coverage is necessary. MRI is ideal for longitudinal studies to examine temporal progression of demyelination and subsequent remyelination in-vivo, and obtaining comprehensive histological data will help to better understand these processes. MRI studies can indicate when and where to focus histological analyses, thus increasing both efficiency and effectiveness of histological comparisons.

To date, few studies have commented on the medio-lateral patterns of demyelination, and its importance is certainly underappreciated. Demyelination is significant in the rostral lateral CC by week 6 and possibly even earlier. We suggest that only through true 4D studies—assessing the changes in the whole brain as a complete 3D organ over time—will we be able to paint a thorough picture of the cuprizone model of demyelination and remyelination. A similar comprehensive study of spatial patterns of demyelination in the chronic state has yet to be completed and will help to answer many of the questions raised here regarding mechanisms of both damage and repair. Understanding the patterns and mechanisms of site-specific damage and repair in the cuprizone model can be helpful in understanding and treating human diseases, such as multiple sclerosis.

The ROI analysis in this study is informative, but has limitations. At first glance it appears that MTR is more sensitive to demyelination in lateral iCC than in lateral sCC when comparing ROI analysis in Fig 3 to BGII results from Table 1. However, two primary factors may obfuscate results from ROI analysis of MRI data in these regions: (1) partial volume effects from surrounding gray matter could artificially decrease MTR at all time points, effectively reducing sensitivity to subtle MTR changes within the CC; (2) enlarged ventricles observed at Week 6 could exaggerate MTR decreases in lateral iCC more than in other regions, giving the appearance of high sensitivity in iCC and no sensitivity in sCC. Lateral CC is quite narrow (2–3 voxels) in both iCC and sCC, thus increasing the potential for partial volume effects in ROI analyses. Although, average MTR maps indicate clear preservation of contrast in all periventricular regions of all slices (Fig 6), which supports our ROI results. Nevertheless, potential for partial volume effects emphasizes importance of high-resolution parametric mapping to demonstrate fine regional variations that may be obscured by ROI analysis alone. Additionally, our BGII quantification approach introduces limits in interpretation of histological results. In some cases only a small number of mounted tissue sections were useable for analysis. Demyelination calculated as percent area as we have done here requires the observer to estimate the extents of CC, which is particularly challenging in heavily demyelinated lateral CC and EC. Limited tissue coverage and biological variation are likely sources of error in myelin content estimation and will contribute to apparent inconsistency in matchup between BGII and MTR values in the narrow lateral extents of the iCC and sCC.

Our results support previous findings that MTR correlates well with demyelination and is a sensitive, if not specific, marker of myelin content in-vivo. We expanded upon previous knowledge of the cuprizone model through a more complete characterization of the spatial pattern of demyelination in the corpus callosum after 6 weeks of cuprizone challenge and demonstrate the importance of appropriate age-matched controls. While the caudal-rostral pattern of demyelination has been shown, this study demonstrates an additional medio-lateral pattern of demyelination in the cuprizone model. This site-specific demyelination may be related to regional gene expression variation and/or spatial proximity to distinct sources of progenitor cells. It is evident that the unique and site-specific demyelination in the cuprizone model has much to teach us about the complex mechanisms of both damage and repair in demyelinating disease.

## References

[1] Carlton W. Studies on the induction of hydrocephalus and spongy degeneration by cuprizone feeding and attempts to antidote the toxicity. Life Sci. 1967;6(1):11–9. 6030552 10.1016/0024-3205(67)90356-6

[2] Carlton W. Response of mice to the chelating agents sodium diethyldithiocarbamate, alpha-benzoinoxime, and biscyclohexanone oxaldihydrazone. Toxicol Appl Pharmacol. 1966;8(3):512–21. 6006739 10.1016/0041-008x(66)90062-7

[3] Lucchinetti C, Brück W, Parisi J, Scheithauer B, Rodriguez M, Lassmann H. Heterogeneity of multiple sclerosis lesions: implications for the pathogenesis of demyelination. Ann Neurol [Internet]. 2000 6;47(6):707–17. Available: http://www.ncbi.nlm.nih.gov/pubmed/10852536. 10.1002/1531-8249(200006)47:6<707::aid-ana3>3.0.co;2-q 10852536

[4] Prineas JW, Parratt JDE. Oligodendrocytes and the early multiple sclerosis lesion. Ann Neurol. 2012;72(1):18–31. 10.1002/ana.23634 22829266

[5] Benetti F, Ventura M, Salmini B, Ceola S, Carbonera D, Mammi S, et al Cuprizone neurotoxicity, copper deficiency and neurodegeneration. Neurotoxicology [Internet]. Elsevier B.V.; 2010 9 [cited 2013 Mar 13];31(5):509–17. Available: http://www.ncbi.nlm.nih.gov/pubmed/20685220. 10.1016/j.neuro.2010.05.008 20685220

[6] Kipp M, Clarner T, Dang J, Copray S, Beyer C. The cuprizone animal model: new insights into an old story. Acta Neuropathol [Internet]. 2009 12 [cited 2014 Sep 16];118(6):723–36. Available: http://www.ncbi.nlm.nih.gov/pubmed/19763593. 10.1007/s00401-009-0591-3 19763593

[7] Goldberg J, Daniel M, Heuvel Y van H, Victor M, Beye C, Clarner T, et al Short-Term Cuprizone Feeding Induces Selective Amino Acid Deprivation with Concomitant Activation of an Integrated Stress Response in Oligodendrocytes. Cell Mol Neurobiol [Internet]. 2013 [cited 2014 Aug 27];33:1087–98. Available: http://link.springer.com/article/10.1007/s10571-013-9975-y. 10.1007/s10571-013-9975-y PMC11497941 23979168

[8] Pasquini L a, Calatayud C a, Bertone Uña a L, Millet V, Pasquini JM, Soto EF. The neurotoxic effect of cuprizone on oligodendrocytes depends on the presence of pro-inflammatory cytokines secreted by microglia. Neurochem Res [Internet]. 2007 2 [cited 2014 Sep 16];32(2):279–92. Available: http://www.ncbi.nlm.nih.gov/pubmed/17063394. 10.1007/s11064-006-9165-0 17063394

[9] Zatta P, Raso M, Zambenedetti P, Wittkowski W, Messori L, Piccioli F, et al Copper and zinc dismetabolism in the mouse brain upon chronic cuprizone treatment. Cell Mol Life Sci [Internet]. 2005 7 [cited 2014 Dec 23];62(13):1502–13. Available: http://www.ncbi.nlm.nih.gov/pubmed/15971002. 10.1007/s00018-005-5073-8 PMC11139106 15971002

[10] Wergeland S, Torkildsen Ø, Myhr K-M, Mørk SJ, Bø L. The cuprizone model: regional heterogeneity of pathology. APMIS [Internet]. 2012 8 [cited 2013 Mar 13];120(8):648–57. Available: http://www.ncbi.nlm.nih.gov/pubmed/22779688 10.1111/j.1600-0463.2012.02882.x 22779688

[11] Steelman AJ, Thompson JP, Li J. Demyelination and remyelination in anatomically distinct regions of the corpus callosum following cuprizone intoxication. Neurosci Res [Internet]. Elsevier Ireland Ltd and Japan Neuroscience Society; 2012 1 [cited 2014 Aug 19];72(1):32–42. Available: http://www.pubmedcentral.nih.gov/articlerender.fcgi?artid=3230728&tool=pmcentrez&rendertype=abstract. 10.1016/j.neures.2011.10.002 PMC3230728 22015947

[12] Fjær S, Bø L, Lundervold A, Myhr K-M, Pavlin T, Torkildsen O, et al Deep gray matter demyelination detected by magnetization transfer ratio in the cuprizone model. PLoS One [Internet]. 2013 1 [cited 2014 Mar 14];8(12):e84162 Available: http://www.pubmedcentral.nih.gov/articlerender.fcgi?artid=3875491&tool=pmcentrez&rendertype=abstract. 10.1371/journal.pone.0084162 PMC3875491 24386344

[13] Zaaraoui W, Deloire M, Merle M, Girard C, Raffard G, Biran M, et al Monitoring demyelination and remyelination by magnetization transfer imaging in the mouse brain at 9.4 T. Magma [Internet]. 2008 [cited 2013 May 14];21(5):357–62. Available: http://link.springer.com/article/10.1007/s10334-008-0141-3 10.1007/s10334-008-0141-3 PMC2598411 18779984

[14] Gudi V, Moharregh-Khiabani D, Skripuletz T, Koutsoudaki PN, Kotsiari A, Skuljec J, et al Regional differences between grey and white matter in cuprizone induced demyelination. Brain Res [Internet]. Elsevier B.V.; 2009 8 4 [cited 2014 Oct 13];1283:127–38. Available: http://www.ncbi.nlm.nih.gov/pubmed/19524552. 10.1016/j.brainres.2009.06.005 19524552

[15] Goldberg J, Clarner T, Beyer C, Kipp M. Anatomical Distribution of Cuprizone-Induced Lesions in C57BL6 Mice. J Mol Neurosci [Internet]. 2015; Available: http://link.springer.com/10.1007/s12031-015-0595-5. 10.1007/s12031-015-0595-5 26067430

[16] Xie M, Tobin JE, Budde MMD, Chen C-I, Trinkaus K, Cross AH, et al Rostro-Caudal Analysis of Corpus Callosum Demyelination and Axon Damage Across Disease Stages Refines Diffusion Tensor Imaging Correlations with Pathological Features. J Neuropathol Exp Neurol [Internet]. 2010 7 [cited 2015 Jan 16];69(7):704–16. Available: http://www.pubmedcentral.nih.gov/articlerender.fcgi?artid=2901930&tool=pmcentrez&rendertype=abstract. 10.1097/NEN.0b013e3181e3de90 PMC2901930 20535036

[17] Tobin J, Xie M, Le T, Song S-K, Arms. Reduced Axonopathy and Enhanced Remyelination Following Chronic Demyelination in Fibroblast Growth Factor-2 (Fgf2) Null Mice: Differential Detection with Diffusion Tensor Imaging. J Neuropathol Exp Neurol [Internet]. 2011 [cited 2014 Oct 9];70(2):157–65. Available: http://www.ncbi.nlm.nih.gov/pmc/articles/PMC3072283/. 10.1097/NEN.0b013e31820937e4 PMC3072283 21343885

[18] Falangola M, Guilfoyle D, Tabesh A, Hui E, Nie X, Jensen J, et al Histological correlation of diffusional kurtosis and white matter modeling metrics in cuprizone‐induced corpus callosum demyelination. NMR Biomed [Internet]. 2014 [cited 2014 Aug 27];27(June 2014):948–57. Available: http://onlinelibrary.wiley.com/doi/10.1002/nbm.3140/full. 10.1002/nbm.3140 PMC5297373 24890981

[19] Merkler D, Boretius S, Stadelmann C, Ernsting T, Michaelis T, Frahm J, et al Multicontrast MRI of remyelination in the central nervous system. NMR Biomed [Internet]. 2005 10 [cited 2013 Jun 6];18(6):395–403. Available: http://www.ncbi.nlm.nih.gov/pubmed/16086436. 10.1002/nbm.972 16086436

[20] Thiessen JD, Zhang Y, Zhang H, Wang L, Buist R, Del Bigio MR, et al Quantitative MRI and ultrastructural examination of the cuprizone mouse model of demyelination. NMR Biomed [Internet]. 2013 11 [cited 2014 Jul 14];26(11):1562–81. Available: http://www.ncbi.nlm.nih.gov/pubmed/23943390. 10.1002/nbm.2992 23943390

[21] Schregel K, Tysiak EW, Garteiser P, Gemeinhardt I, Prozorovski T, Aktas O, et al Demyelination reduces brain parenchymal stiffness quantified in vivo by magnetic resonance elastography. PNAS [Internet]. 2012 [cited 2014 Jan 9];109(17):6650–5. Available: http://www.pnas.org/content/109/17/6650.short. 10.1073/pnas.1200151109 PMC3340071 22492966

[22] Vavasour IM, Laule C, Li DKB, Traboulsee AL, MacKay AL. Is the magnetization transfer ratio a marker for myelin in multiple sclerosis? J Magn Reson Imaging [Internet]. 2011 3 [cited 2014 Aug 27];33(3):713–8. Available: http://www.ncbi.nlm.nih.gov/pubmed/21563257. 10.1002/jmri.22441 21563257

[23] Wu Q-Z, Yang Q, Cate HS, Kemper D, Binder M, Wang H-X, et al MRI identification of the rostral-caudal pattern of pathology within the corpus callosum in the cuprizone mouse model. J Magn Reson Imaging [Internet]. 2008 3 [cited 2013 May 8];27(3):446–53. Available: http://www.ncbi.nlm.nih.gov/pubmed/17968901. 10.1002/jmri.21111 17968901

[24] Boretius S, Escher A, Dallenga T, Wrzos C, Tammer R, Brück W, et al Assessment of lesion pathology in a new animal model of MS by multiparametric MRI and DTI. Neuroimage [Internet]. Elsevier Inc.; 2012 2 1 [cited 2014 Dec 23];59(3):2678–88. Available: http://www.ncbi.nlm.nih.gov/pubmed/21914485. 10.1016/j.neuroimage.2011.08.051 21914485

[25] Gudi V, Gingele S, Skripuletz T, Stangel M. Glial response during cuprizone-induced de- and remyelination in the CNS: lessons learned. Front Cell Neurosci [Internet]. 2014 1 [cited 2014 Aug 18];8(March):73 Available: http://www.pubmedcentral.nih.gov/articlerender.fcgi?artid=3952085&tool=pmcentrez&rendertype=abstract. 10.3389/fncel.2014.00073 PMC3952085 24659953

[26] Skripuletz T, Bussmann J-H, Gudi V, Koutsoudaki PN, Pul R, Moharregh-Khiabani D, et al Cerebellar cortical demyelination in the murine cuprizone model. Brain Pathol [Internet]. 2010 3 [cited 2013 Mar 13];20(2):301–12. Available: http://www.ncbi.nlm.nih.gov/pubmed/19371354. 10.1111/j.1750-3639.2009.00271.x PMC8094790 19371354

[27] Schmidt T, Awad H, Slowik a, Beyer C, Kipp M, Clarner T. Regional heterogeneity of cuprizone-induced demyelination: topographical aspects of the midline of the corpus callosum. J Mol Neurosci [Internet]. 2013 1 [cited 2013 Apr 30];49(1):80–8. Available: http://www.ncbi.nlm.nih.gov/pubmed/23054589. 10.1007/s12031-012-9896-0 23054589

[28] Yarnykh VL. Fast macromolecular proton fraction mapping from a single off-resonance magnetization transfer measurement. Magn Reson Med. 2012;68(1):166–78. 10.1002/mrm.23224 22190042 PMC3311766

[29] Sled JG, Pike GB. Quantitative imaging of magnetization transfer exchange and relaxation properties in vivo using MRI. Magn Reson Med. 2001;46(5):923–31. 11675644 10.1002/mrm.1278

[30] Tardif CL, Bedell BJ, Eskildsen SF, Collins DL, Pike GB. Quantitative magnetic resonance imaging of cortical multiple sclerosis pathology. Mult Scler Int. 2012;2012:742018 10.1155/2012/742018 23213531 PMC3506905

[31] Portnoy S, Stanisz GJ. Modeling pulsed magnetization transfer. Magn Reson Med. 2007;58(1):144–55. 17659607 10.1002/mrm.21244

[32] Stanisz GJ, Odrobina EE, Pun J, Escaravage M, Graham SJ, Bronskill MJ, et al T1, T2 relaxation and magnetization transfer in tissue at 3T. Magn Reson Med. 2005;54(3):507–12. 16086319 10.1002/mrm.20605

[33] Van Rossum G. Python programming language. USENIX Annual Technical Conference. 2007.

[34] Jenkinson M, Beckmann CF, Behrens TEJ, Woolrich MW, Smith SM. Fsl. Neuroimage [Internet]. 2012 8 15 [cited 2013 Sep 20];62(2):782–90. Available: http://www.ncbi.nlm.nih.gov/pubmed/21979382. 10.1016/j.neuroimage.2011.09.015 21979382

[35] Smith SM, Nichols TE. Threshold-free cluster enhancement: addressing problems of smoothing, threshold dependence and localisation in cluster inference. Neuroimage [Internet]. Elsevier Inc.; 2009 1 1 [cited 2014 Jul 10];44(1):83–98. Available: http://www.ncbi.nlm.nih.gov/pubmed/18501637. 10.1016/j.neuroimage.2008.03.061 18501637

[36] Allen Institute for Brain Science. Allen Mouse Brain Atlas. http://MouseBrain-MapOrg/ [Internet]. 2014;(November):1–26. Available: http://atlas.brain-map.org/atlas?atlas=1#atlas=1&plate=100960224&structure=549&x=5280.1904296875&y=3744.0000697544647&zoom=-3&resolution=11.97&z=5.

[37] Lein ES, Hawrylycz MJ, Ao N, Ayres M, Bensinger A, Bernard A, et al Genome-wide atlas of gene expression in the adult mouse brain. Nature [Internet]. 2007 1 11 [cited 2014 Jul 9];445(7124):168–76. Available: http://www.ncbi.nlm.nih.gov/pubmed/17151600. 10.1038/nature05453 17151600

[38] Stidworthy MF, Genoud S, Suter U, Mantei N, Franklin RJM. Quantifying the early stages of remyelination following cuprizone-induced demyelination. Brain Pathol [Internet]. 2003 7;13(3):329–39. Available: http://www.ncbi.nlm.nih.gov/pubmed/12946022. 10.1111/j.1750-3639.2003.tb00032.x PMC8095752 12946022

[39] Hiremath MM, Saito Y, Knapp GW, Ting JP, Suzuki K, Matsushima GK. Microglial/macrophage accumulation during cuprizone-induced demyelination in C57BL/6 mice. J Neuroimmunol [Internet]. 1998 12 1;92(1–2):38–49. Available: http://www.ncbi.nlm.nih.gov/pubmed/9916878. 10.1016/s0165-5728(98)00168-4 9916878

[40] Acs P, Kalman B. Autoimmunity [Internet] Second. Perl A, editor. Archives of Pathology & Laboratory Medicine. Totowa, NJ: Humana Press; 2012 [cited 2014 Sep 16]. 403–431 p. Available: http://www.archivesofpathology.org/doi/full/10.1043/1543-2165(2006)130[409b:AMAP]2.0.CO;2.

[41] Yang H-J, Wang H, Zhang Y, Xiao L, Clough RW, Browning R, et al Region-specific susceptibilities to cuprizone-induced lesions in the mouse forebrain: Implications for the pathophysiology of schizophrenia Brain Res [Internet]. Elsevier B.V.; 2009 5 13 [cited 2014 Oct 8];1270:121–30. Available: http://www.ncbi.nlm.nih.gov/pubmed/19306847. 10.1016/j.brainres.2009.03.011 19306847

[42] Allen Institute for Brain Science. Allen Mouse Brain Atlas [Internet] [Internet]. 2015. Available: http://connectivity.brain-map.org.

[43] Mottershead JP, Schmierer K, Clemence M, Thornton JS, Scaravilli F, Barker GJ, et al High field MRI correlates of myelin content and axonal density in multiple sclerosis: A post-mortem study of the spinal cord. J Neurol. 2003;250(11):1293–301. 14648144 10.1007/s00415-003-0192-3

[44] Sturrock R. Myelination of the mouse corpus callosum. Neuropathol Appl Neurobiol [Internet]. 1980 [cited 2014 Oct 10];6(6):415–20. Available: http://onlinelibrary.wiley.com/doi/10.1111/j.1365-2990.1980.tb00219.x/abstract. 10.1111/j.1365-2990.1980.tb00219.x 7453945

[45] Mason JL, Langaman C, Morell P, Suzuki K, Matsushima GK. Episodic demyelination and subsequent remyelination within the murine central nervous system: changes in axonal calibre. Neuropathol Appl Neurobiol [Internet]. 2001 2;27(1):50–8. Available: http://doi.wiley.com/10.1046/j.0305-1846.2001.00301.x. 10.1046/j.0305-1846.2001.00301.x 11299002

[46] Aboitiz F, Scheibel AB, Fisher RS, Zaidel E. Fiber composition of the human corpus callosum. Brain Res [Internet]. 1992 12;598(1–2):143–53. Available: http://linkinghub.elsevier.com/retrieve/pii/000689939290178C. 10.1016/0006-8993(92)90178-c 1486477

[47] Aboitiz F, Montiel J. One hundred million years of interhemispheric communication: the history of the corpus callosum. Braz J Med Bio Res. 2003;36:409–20. 12700818 10.1590/s0100-879x2003000400002

[48] Guglielmetti C, Veraart J, Roelant E, Mai Z, Daans J, Van Audekerke J, et al Diffusion kurtosis imaging probes cortical alterations and white matter pathology following cuprizone-induced demyelination and spontaneous remyelination. Neuroimage [Internet]. Elsevier B.V.; 2016;125:363–77. Available: 10.1016/j.neuroimage.2015.10.052 PMC4935929 26525654

[49] Jarjour A a, Kennedy TE. Oligodendrocyte precursors on the move: mechanisms directing migration. Neuroscientist [Internet]. 2004 4 [cited 2014 Dec 9];10(2):99–105. Available: http://www.ncbi.nlm.nih.gov/pubmed/15070484. 10.1177/1073858403260751 15070484

[50] Cate HS, Sabo JK, Merlo D, Kemper D, Aumann TD, Robinson J, et al Modulation of bone morphogenic protein signalling alters numbers of astrocytes and oligodendroglia in the subventricular zone during cuprizone-induced demyelination. J Neurochem [Internet]. 2010 10 [cited 2014 Oct 16];115(1):11–22. Available: http://www.ncbi.nlm.nih.gov/pubmed/20193041. 10.1111/j.1471-4159.2010.06660.x 20193041

[51] Back SA, Rosenberg PA. Pathophysiology of Glia in Perinatal White Matter Injury. 2014 1790–1815 p. 10.1002/glia.22658 PMC4163108 24687630

[52] Kaneko N, Kako E, Sawamoto K. Prospects and limitations of using endogenous neural stem cells for brain regeneration. Genes (Basel) [Internet]. 2011 1 [cited 2014 Dec 4];2(1):107–30. Available: http://www.pubmedcentral.nih.gov/articlerender.fcgi?artid=3924842&tool=pmcentrez&rendertype=abstract. 10.3390/genes2010107 PMC3924842 24710140

[53] Donegà M, Giusto E, Cossetti C, Pluchino S. Systemic neural stem cell-based therapeutic interventions for inflammatory CNS disorders Neural Stem Cells—New Perspect [Internet]. 2013 [cited 2014 Dec 4]; Available: http://www.intechopen.com/books/neural-stem-cells-new-perspectives/systemic-neural-stem-cell-based-therapeutic-interventions-for-inflammatory-cns-disorders.

[54] Mason JL, Jones JJ, Taniike M, Morell P, Suzuki K, Matsushima GK. Mature Oligodendrocyte Apoptosis Precedes IGF-1 Production and Oligodendrocyte Progenitor Accumulation and Differentiation During Demyelination / Remyelination. J Neurosci Res [Internet]. 2000;61:251–62. Available: http://onlinelibrary.wiley.com/doi/10.1002/1097-4547(20000801)61:3<251::AID-JNR3>3.0.CO;2-W/pdf. 10.1002/1097-4547(20000801)61:3<251::AID-JNR3>3.0.CO;2-W 10900072

[55] Petratos S, Gonzales MF, Azari MF, Marriott M, Minichiello R a, Shipham K a, et al Expression of the low-affinity neurotrophin receptor, p75(NTR), is upregulated by oligodendroglial progenitors adjacent to the subventricular zone in response to demyelination. Glia [Internet]. 2004 10 [cited 2014 Dec 11];48(1):64–75. Available: http://www.ncbi.nlm.nih.gov/pubmed/15326616. 10.1002/glia.20056 15326616

[56] Guglielmetti C, Praet J, Rangarajan JR, Vreys R, De Vocht N, Maes F, et al Multimodal imaging of subventricular zone neural stem/progenitor cells in the cuprizone mouse model reveals increased neurogenic potential for the olfactory bulb pathway, but no contribution to remyelination of the corpus callosum. Neuroimage [Internet]. Elsevier B.V.; 2014 2 1 [cited 2014 Nov 17];86:99–110. Available: http://www.ncbi.nlm.nih.gov/pubmed/23933305. 10.1016/j.neuroimage.2013.07.080 23933305

[57] Sawamoto K, Wichterle H, Gonzalez-Perez O, Cholfin JA, Yamada M, Spassky N, et al New neurons follow the flow of cerebrospinal fluid in the adult brain. Science (80-) [Internet]. 2006 [cited 2014 Dec 10];311(5761):629–32. Available: http://www.sciencemag.org/content/311/5761/629.short. 10.1126/science.1119133 16410488

[58] Beatrice B, Magalon K, Pascale D, Cayre M. Region and dynamic specificities of adult neural stem cells and oligodendrocyte precursors in myelin regeneration in the mouse brain. Biol Open [Internet]. 2015;1–13. Available: http://bio.biologists.org/cgi/doi/10.1242/bio.012773. 10.1242/bio.012773 PMC4542288 26142314

[59] Silvestroff L, Bartucci S, Soto E, Gallo V, Pasquini J, Franco P. Cuprizone-Induced demyelination in CNP::GFP transgenic mice. J Comp Neurol. 2010;518(12):2261–83. 10.1002/cne.22330 20437527

[60] Baloch S, Verma R, Huang H, Khurd P, Clark S, Yarowsky P, et al Quantification of brain maturation and growth patterns in C57BL/6J mice via computational neuroanatomy of diffusion tensor images. Cereb Cortex [Internet]. 2009 3 [cited 2014 Oct 10];19(3):675–87. Available: http://www.pubmedcentral.nih.gov/articlerender.fcgi?artid=3140198&tool=pmcentrez&rendertype=abstract. 10.1093/cercor/bhn112 PMC3140198 18653668

[61] Larvaron P, Boespflug-tanguy O, Renou J, Bonny J. In vivo analysis of the post-natal development of normal mouse brain by DTI. NMR Biomed. 2007;20:413–21. 17120295 10.1002/nbm.1082

